# Assessment of *Cytospora* Isolates From Conifer Cankers in China, With the Descriptions of Four New *Cytospora* Species

**DOI:** 10.3389/fpls.2021.636460

**Published:** 2021-02-18

**Authors:** Meng Pan, Haiyan Zhu, Chengming Tian, Manrong Huang, Xinlei Fan

**Affiliations:** ^1^The Key Laboratory for Silviculture and Conservation of Ministry of Education, Beijing Forestry University, Beijing, China; ^2^Beijing Museum of Natural History, Beijing, China

**Keywords:** canker disease, coniferous trees, pathogen, phylogeny, taxonomy

## Abstract

*Cytospora* species are widely distributed and often occur as endophytes, saprobes or phytopathogens. They primarily cause canker and dieback diseases of woody host plants, leading to the growth weakness or death of host plants, thereby causing significant economic and ecological losses. In order to reveal the diversity of *Cytospora* species associated with canker and dieback diseases of coniferous trees in China, we assessed 11 *Cytospora* spp. represented by 28 fungal strains from symptomatic branches or twigs of coniferous trees, i.e., *Juniperus procumbens, J. przewalskii, Picea crassifolia, Pinus armandii, P. bungeana, Platycladus orientalis* in China. Through morphological observations and multilocus phylogeny of ITS, LSU, *act, rpb2, tef1-*α, and *tub2* gene sequences, we focused on four novel *Cytospora* species (*C. albodisca, C. discostoma, C. donglingensis*, and *C. verrucosa*) associated with *Platycladus orientalis*. This study represented the first attempt to clarify the taxonomy of *Cytospora* species associated with canker and dieback symptoms of coniferous trees in China.

## Introduction

Coniferous trees are excellent landscaping species with a high ornamental and economic value. They are widely distributed as evergreen coniferous tree species and cultivated throughout China, except in Xinjiang and Qinghai Provinces (Ming, 2016). However, several coniferous trees are threatened by various pathogens in the process of planting and cultivation. Fan et al. (2020) reported five novel and one known *Cytospora* species causing canker and dieback diseases in conifers, with detailed descriptions and illustrations. *Armillaria* spp., *Heterobasidion annosum*, and *Phellinus* spp. have been reported to cause root and butt rot (Shaw and Kile, 1991; Hansen and Goheen, 2000). Moreover, leaf blight and *Phytophthora* diseases (Tucker and Milbrath, 1942; Phillips and Burdekin, 1992; Schlenzig et al., 2014) are also destructive to conifers.

*Cytospora* is one of the most important pathogenic fungi of hardwoods and coniferous trees with a worldwide distribution and large host range (Adams et al., 2005, 2006; Fan et al., 2014a,b, 2015a,b; Ariyawansa et al., 2015; Liu et al., 2015; Maharachchikumbura et al., 2015, 2016; Hyde et al., 2016; Li et al., 2016; Lawrence et al., 2017, 2018; Norphanphoun et al., 2017, 2018). Dieback and stem canker caused by *Cytospora* leads to the growth weakness or death of host plants, thereby causing significant economic and ecological losses (Adams et al., 2005). In conifers, *Cytospora* canker commonly occurs in the lowermost branches of mature trees, and stops spreading at the trunk (Adams et al., 2005). The asexual morph of *Cytospora* is characterized by the pycnidial stromata immersed in the bark with a single or multiple locule(s), with or without conceptacle. The conidia are aseptate, hyaline, allantoid, eguttulate, and smooth (Adams et al., 2005). The sexual morph is characterized by the ascomata immersed in the substrate with an erumpent pseudostroma, with or without necks. Asci are unitunicate, clavate to cylindrical. Ascospores are biseriate or multi-seriate, elongate-allantoid, thin-walled, hyaline, aseptate (Adams et al., 2005).

The taxonomy of the genus *Cytospora* is rather confusing. Ehrenberg (1818) established *Cytospora* and described four species simultaneously. Eighteen *Cytospora* species were proposed by Fries (1823), but the genus was recorded as *Cytispora* due to a misspelling. Thereafter, Saccardo (1884) revised the name to *Cytospora* and introduced 144 species. Due to the controversy in the corresponding relationship between sexual and asexual morphs, there were several synonyms, which had caused difficulties in the identification of *Cytospora* (Adams et al., 2005). Adams et al. (2005) officially reported that the sexual genera *Leucocytospora, Leucostoma, Valsella*, and *Valseutypella* are synonyms of *Valsa*. The traditional identification of *Cytospora* species is based heavily on their host affiliations. Nevertheless, the species occurrence may be related to geographical and environmental factors rather than host specificity (Fan et al., 2014a,b; Fan et al., 2015a,b). To more accurately identify *Cytospora* species, several species have been described based on morphological observations and multilocus phylogeny in recent studies (Yang et al., 2015; Lawrence et al., 2017, 2018; Zhu et al., 2018, 2020; Fan et al., 2020; Jiang et al., 2020; Shang et al., 2020). Norphanphoun et al. (2017) used four loci to describe 14 new species isolated from *Rosa, Salix*, and *Sorbus*. Lawrence et al. (2018) reported 15 *Cytospora* species that infected fruit trees and crops using a multiphasic approach. Pan et al. (2020) assessed 23 species of *Cytospora* associated with canker and dieback disease of Rosaceae members in China by six-locus phylogeny.

There are only a few relative taxonomic studies of *Cytospora* canker or dieback disease of conifers. Thus, there is an urgent need for studies to clarify the pathogens causing dieback and stem canker in coniferous trees. In this study, we aimed to reveal the diversity of *Cytospora* species associated with canker and dieback diseases of coniferous trees in China. As a part of an investigation of pathogens that cause canker or dieback disease in China, 28 *Cytospora* strains in coniferous trees with obvious symptoms were evaluated. Morphological characters in conjunction with multilocus phylogenetic analyses provided valuable information to identify the phylogenetic position of these isolates. Herein, we also introduced *Cytospora albodisca, C. discostoma, C. donglingensis*, and *C. verrucosa* as four new species with descriptions and illustrations, and compared them with other species in the genus.

## Materials and Methods

### Sample Collection and Isolation

Fresh specimens with typical *Cytospora* fruiting bodies were collected from the infected twigs and branches of coniferous trees during collecting trips in China. A total of 12 isolates were obtained by removing a mucoid spore mass from conidiomata on the twigs and branches, spreading the suspension over the surface with standard potato dextrose agar (PDA) in a Petri dish, and incubating at 25°C for up to 24 h. Single germinating conidia were transferred on to fresh PDA plates. All specimens and isolates were deposited in the Beijing Museum of Natural History (BJM) and the working Collection of X.L. Fan (CF) housed in Beijing Forestry University (BJFU). Axenic living cultures were deposited at China Forestry Culture Collection Centre (CFCC).

### Morphological Analyses

Species identification was based on morphological characteristics of the ascomata or conidiomata produced on infected host materials. The macro-morphological characteristics including structure and size of stromata; the size, color, and shape of discs; number and diameter of ostioles per disc; presence and absence of conceptacle were determined under a Leica stereomicroscope (M205). The micro-morphological characteristics including size and shape of conidiophores and conidia were determined under a Nikon Eclipse 80i microscope equipped with a Nikon digital sight DS-Ri2 high-definition color camera with differential interference contrast (DIC). Over 10 ascomata/conidiomata were sectioned, and 10 asci and 30 ascospores/conidia were selected randomly for measurement. Colony morphology and growth rates were recorded and colony colors were described after 1 or 2 weeks according to the color charts of Rayner (1970). Adobe Bridge CS v.6 and Adobe Photoshop CS v.5 were used for the manual editing. Taxonomic novelties and descriptions were deposited in MycoBank (Crous et al., 2004).

### DNA Extraction and PCR Amplification

Genomic DNA was extracted using the modified CTAB method (Doyle and Doyle, 1990) from mycelium which was cultured on PDA with cellophane and obtained from the surface of cellophane by scraping. The extracted DNA were estimated visually by electrophoresis in 1% agarose gels by comparing band intensity with a DNA marker 1 kbp (Takara Biotech). The qualities of DNA were measured with a NanoDrop^TM^ 2000 (Thermo, USA). Six loci including the internal transcribed spacer (ITS), the large nuclear ribosomal RNA subunit (LSU), the partial actin (*act*), the RNA polymerase II subunit (*rpb2*), the translation elongation factor 1-α (*tef1-*α), and the beta-tubulin (*tub2*) genes were amplified and sequenced using the primer pairs ITS1 and ITS4 (White et al., 1990), LROR and LR7 (Vilgalys and Hester, 1990), ACT-512F and ACT-783R (Carbone and Kohn, 1999), RPB2-5F and fRPB2-7cR (Liu et al., 1999), EF-688F and EF-1251R (Alves et al., 2008), and Bt-2a and Bt-2b (Glass and Donaldson, 1995). The PCR amplicons were electrophoresed in 2% agarose gels. DNA sequencing was carried out using an ABI PRISM® 3730XL DNA Analyzer with BigDye® Terminater Kit v.3.1 (Invitrogen) at the Shanghai Invitrogen Biological Technology Company Limited (Beijing, China). DNA sequences generated by the forward and reverse primers were used to obtain consensus sequences using Seqman v.9.0.4 (DNASTAR Inc., Madison, WI, USA).

### Phylogenetic Analyses

The sequences generated from this study were analyzed with related *Cytospora* taxa which were obtained from GenBank and recent publications (Supplementary Table 1). To infer their phylogenetic relationship for the new sequences, the alignment based on ITS, LSU, *act, rpb2, tef1-*α, and *tub2* sequence data was performed using MAFFT v.6 (Katoh and Standley, 2013) and edited manually using MEGA v.6.0 (Tamura et al., 2013). For individual sequences, some characters were excluded from both ends of the alignments to approximate the size of our sequences. The sequences of *Diaporthe vaccinii* (CBS 160.32) was included as outgroup in all analyses. Phylogenetic analyses were performed with PAUP v.4.0b10 for maximum parsimony (MP) (Swofford, 2003), MrBayes v.3.1.2 for Bayesian Inference (BI) (Ronquist and Huelsenbeck, 2003), and PhyML v.3.0 for maximum likelihood (ML) (Guindon et al., 2010).

MP analysis in PAUP v.4.0b10 was conducted using a heuristic search option of 1,000 random-addition sequences with tree-bisection-reconnection (TBR) as the branch-swapping algorithm (Swofford, 2003). The branches of zero length were collapsed using the command minbrlen, and all equally parsimonious trees were saved. Clade stability was assessed using a bootstrap (BT) analysis of 1,000 replicates (Hillis and Bull, 1993). Tree length (TL), consistency index (CI), retention index (RI), and rescaled consistency (RC) were calculated for all equally parsimonious trees. ML analysis in PhyML v.3.0 was performed with a general time reversible model (GTR) of site substitution following previous studies (Fan et al., 2020), including estimation of gamma-distributed rate heterogeneity and a proportion of invariant sites (Guindon et al., 2010). An evolutionary model for BI was estimated independently for each locus using MrModeltest v.2.3. The best-fit model was selected under the Akaike Information Criterion (AIC) (Posada and Crandall, 1998). BI analysis in MrBayes v.3.1.2 was done by a Markov Chain Monte Carlo (MCMC) algorithm with Bayesian posterior probabilities (BPP) (Rannala and Yang, 1996). Two MCMC chains started from random trees for 10 million generations, and trees were sampled each 100th generations. The first 25% of trees were discarded as the burn-in phase of each analysis, BPP were calculated to assess the remaining 7,500 trees (Rannala and Yang, 1996). Phylogram was viewed in Figtree v.1.3.1 (Rambaut and Drummond, 2010). All novel sequences derived from this study data were deposited in GenBank. The multigene sequence alignment files were deposited in TreeBASE (www.treebase.org; accession number: S27070).

## Results

**Figure 1:**
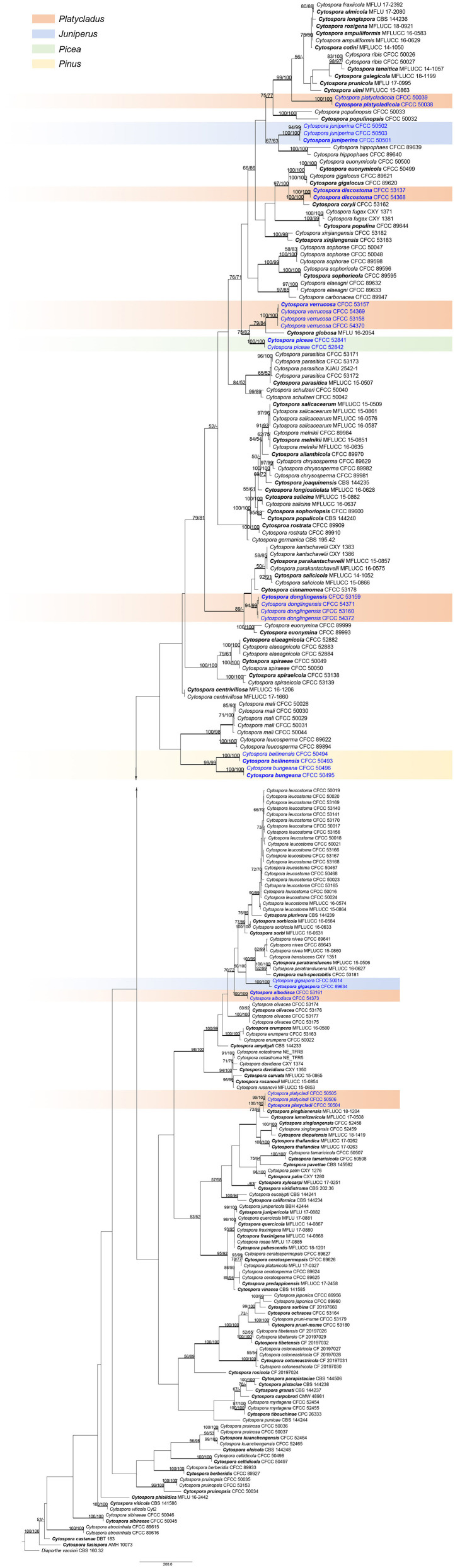
Phylogram of *Cytospora* based on combined ITS, LSU, *act, rpb2, tef1-*α, and *tub2* genes. MP and ML bootstrap support values above 50% are shown at the first and second position. Thickened branches represent posterior probabilities above 0.95 from BI. Ex-type strains are in bold. Strains in current study are in blue.

A total of 12 *Cytospora* specimens were collected from symptomatic coniferous trees in China. The combined alignments matrix (ITS, LSU, *act, rpb2, tef1-*α, and *tub2*) was used to clarify the phylogenetic position of these *Cytospora* species. Following alignment, the sequences for six loci comprised 231 *Cytospora* strains with *Diaporthe vaccinii* CBS 160.32 as the outgroup strain. The aligned matrix comprised 3,761 characters including gaps, of which 2,011 characters were constant, 258 variable characters were parsimony-uninformative, and 1,492 characters were variable and parsimony-informative. MP analysis generated 200 equally parsimonious trees with similar clade topologies, and one of which is presented in Figure 1 (TL = 10402, CI = 0.300, RI = 0.799, RC = 0.239). For BI analyses, the best-fit model of nucleotide evolution was deduced on the AIC (ITS and *act*: GTR+I+G; LSU: TrN+I+G; *rpb2* and *tef1-*α: TrN+I+G; *tub2*: HKY+I+G). ML and Bayesian analyses did not significantly differ from MP tree. The MP bootstrap supports (MP-BS) and ML bootstrap (ML-BS) equal to or above 50% were shown in branches in Figure 1. The branches with significant Bayesian posterior probabilities (BPP) equal to or above 0.95 were thickened in the phylogram. Based on the multigene phylogeny and morphology, the current 12 strains clustered in four clades were equivalent to four *Cytospora* species, were herein described as *C. albodisca, C. discostoma, C. donglingensis*, and *C. verrucosa*. All detailed descriptions and notes are below.

### Taxonomy

**Figure 2:**
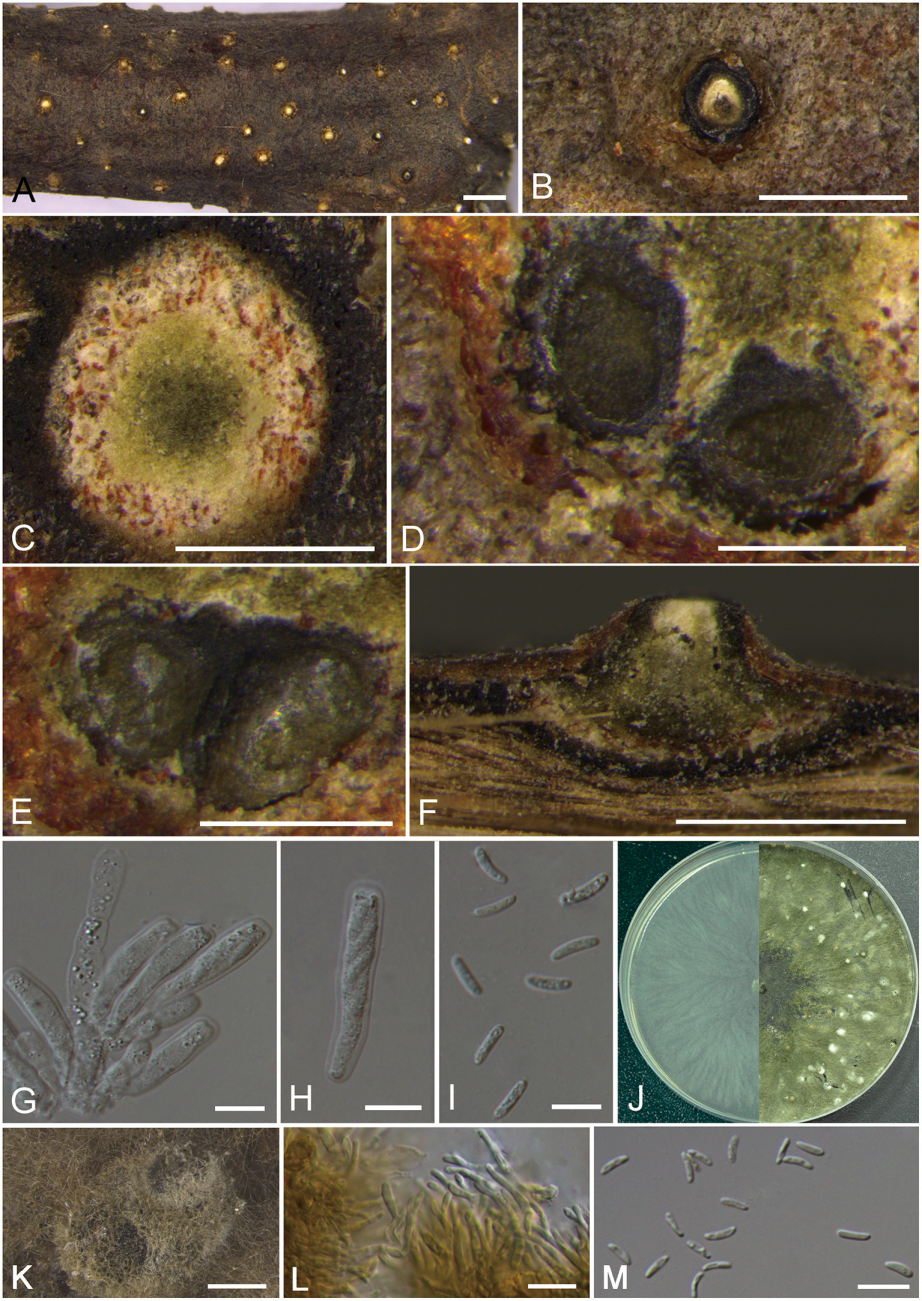
Morphology of *Cytospora albodisca* (BJFC CF2019908). **(A–C)** Habit of ascomata on twig. **(D,E)** Transverse section of ascomata. **(F)** Longitudinal section through ascomata. **(G)** Asci and ascospores. **(H)** Ascus. **(I)** Ascospores. **(J)** Colonies on PDA at 3 days (left) and 30 days (right). **(K)** Conidiomata from culture on PDA. **(L)** Conidiophores. **(M)** Conidia. Bars: **(A)** = 1 mm; **(B–D), (F)** = 250 μm; **(E,K)** = 500 μm; **(F,G), (L,M)** = 10 μm.

***Cytospora albodisca*** M. Pan & X.L. Fan, sp. nov. Figure 2

MycoBank MB 837629

*Typification*: CHINA. Beijing City: Mentougou District, Mount Dongling, Xiaolongmen Forestry Centre, 115°28′28.52″E, 39°55′49.42″N, from branches of *Platycladus orientalis*, 17 August 2017, *H.Y. Zhu* & *X.L. Fan* (**holotype** BJFC CF2019908, **isotype** BJM 240516), ex-type living culture CFCC 53161.

*Etymology*: Named after the white disc of ascostromata.

*Descriptions*: Sexual morph: Ascostromata immersed in the bark, erumpent through the surface of bark, scattered, immature, 580–910 μm in diam. Conceptacle present. Ectostromatic disc beige to orange, circular, surrounded by dark ectostromatic tissue, with single ostiole in a disc, 220–375 μm in diam. Ostioles single, white to pale yellow, at the same or below level as the disc, 90–150 μm in diam. Perithecia beige with a little black when mature, flask-shaped to spherical, arranged irrugularly, 340–550 μm in diam. Asci free, clavate to elongate-obovoid, 30–35 × 6.5–8 μm, 8-spored. Ascospores uniserial, elongate-allantoid, thin-walled, hyaline, aseptate, slightly rough, 8–14 × 2–3.5 (av. = 11.1 ± 2.6 × 2.6 ± 0.3, *n* = 30) μm. Asexual morph: On PDA, pycnidial stromata covered by abundant aerial mycelium, globose, solitary or aggregated deeply embedded in the medium, erumpent, dark green, 780–1,170 μm in diam. Conidiophores cylindrical, thinner in the middle than the two ends, hyaline, unbranched, straight to slightly sinuous, 10.5–20.5 × 1.5–2.5 μm. Conidiogenous cells enteroblastic, phialidic. Conidia hyaline, allantoid, occasionally with little curve, rough, aseptate, occasionally biguttulate, 5–7 × 1–2 (av. = 5.6 ± 0.5 × 1.4 ± 0.2, *n* = 30) μm.

*Culture characteristics*: Cultures on PDA are initially white, growing fast up to 7 cm in diam. after 3 days and entirely covering the 9 cm Petri dish after 5 days, becoming dark herbage green to dull green after 7–10 days. Colonies are sparse in the center and compact to the margin, felt-like. Pycnidia distributed irregularly on surface.

*Additional material examined*: CHINA. Beijing City: Mentougou District, Mount Dongling, Xiaolongmen Forestry Centre, 115°27′05.00″E, 39°59′23.58″N, from branches of *Platycladus orientalis*, 17 August 2017, *H.Y. Zhu* & *X.L. Fan* (BJFC CF20201008), living culture CFCC 54373.

*Notes*: *Cytospora albodisca* is associated with canker disease of *Platycladus orientalis* in current study. It can be identified by having ascostroma surrounded by a black conceptacle, producing allantoid, aseptate ascospores (8–14 × 2–3.5 μm). Further, the two strains are phylogenetically separated from all other available strains included in this study. Based on DNA sequence data and morphology, therefore, we describe this species as new.

**Figure 3:**
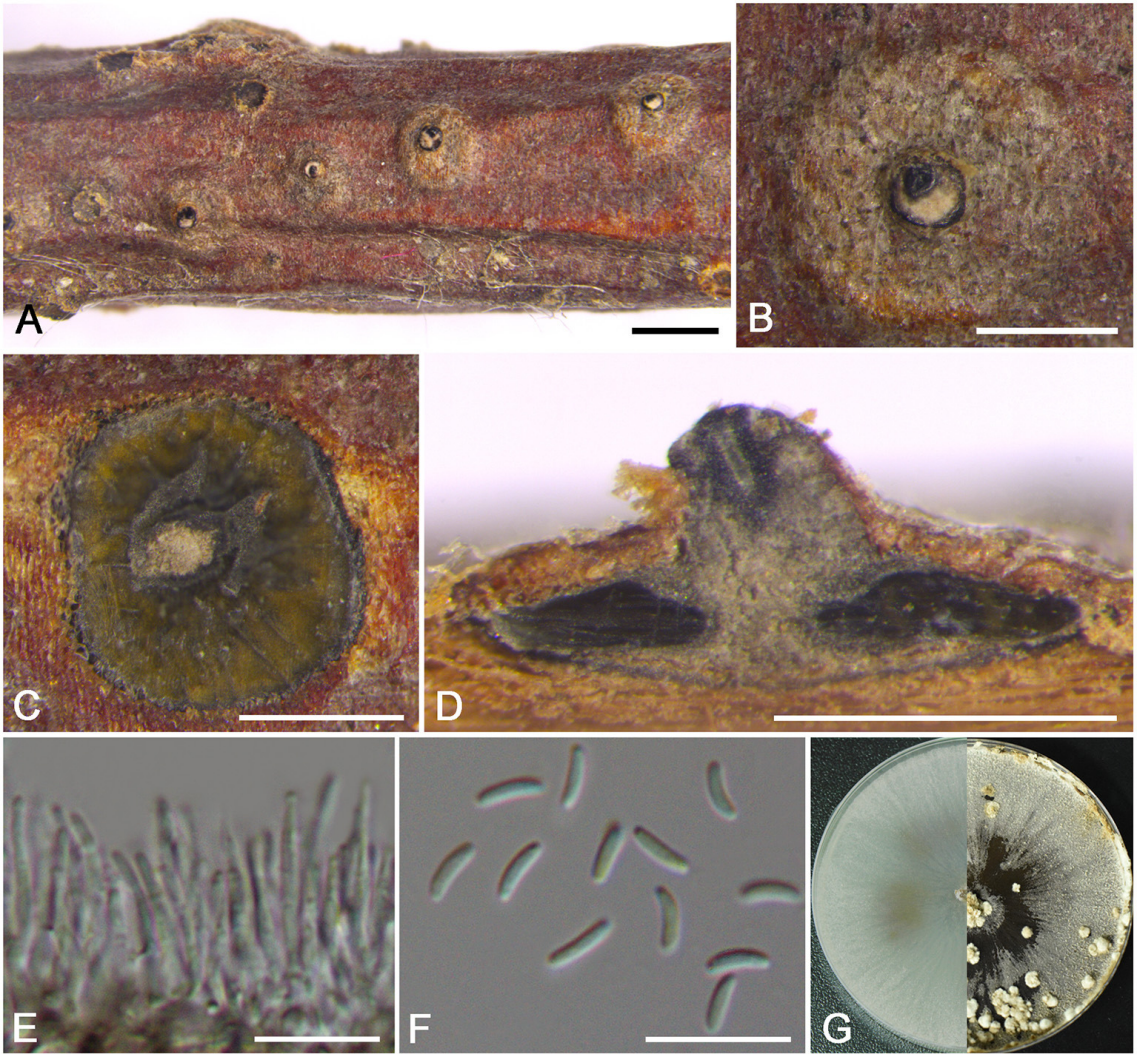
Morphology of *Cytospora discostoma* (BJFC CF2019802). **(A,B)** Habit of conidiomata on twig. **(C)** Transverse section of conidioma. **(D)** Longitudinal section through conidioma. **(E)** Conidiophores and conidiogenous cells. **(F)** Conidia. **(G)** Colonies on PDA at 3 days (left) and 30 days (right). Bars: **(A)** = 1 mm; **(B–D)** = 500 μm; **(E,F)** = 10 μm.

***Cytospora discostoma*** M. Pan & X.L. Fan, sp. nov. Figure 3

MycoBank MB 837632

*Typification*: CHINA. Beijing City: Mentougou District, Mount Dongling, Xiaolongmen Forestry Centre, 115°28′25.47″E, 39°56′48.57″N, from branches of *Platycladus orientalis*, 17 August 2017, *H.Y. Zhu* & *X.L. Fan* (**holotype** BJFC CF2019802, **isotype** BJM 240517), ex-type living culture CFCC 53137.

*Etymology*: Named after the distinct disc of stromata on branches.

*Descriptions*: Sexual morph: not observed. Asexual morph: Pycnidial stromata discoid, immersed in the bark, scattered, erumpent through the surface of bark in a large area, with multiple locules and conspicuous central column. Central column beneath the disc more or less conical, pale gray. Conceptacle present. Ectostromatic disc gray to black, discoid, circular to ovoid, 210–320 μm in diam., with a single ostiole per disc. Ostiole gray to black, nearly at the same level as the disc surface, 90–115 μm in diam. Locules numerous, subdivided frequently by invaginations with common walls, circular to ovoid, 965–1050 μm in diam. Conidiophores hyaline, unbranched, approximately cylindrical, 14–18 × 1–1.5 μm. Conidiogenous cells enteroblastic, phialidic. Conidia hyaline, elongate-allantoid, smooth, aseptate, 4.5–5.5 × 1–1.5 (av. = 4.9 ± 0.3 × 1.2 ± 0.1, *n* = 30) μm.

*Culture characteristics*: Cultures on PDA are initially white with hazel in the center, growing fast up to cover the 9 cm Petri dish after 3 days, becoming brown vinaceous after 7–10 days. Colonies are flat with a uniform texture. Pycnidia distributed irregularly on surface.

*Additional material examined*: CHINA. Beijing City: Mentougou District, Mount Dongling, Xiaolongmen Forestry Centre, 115°26′51.27″E, 39°58′19.62″N, from branches of *Platycladus orientalis*, 17 August 2017, *H.Y. Zhu* & *X.L. Fan* (BJFC CF20201002), living culture CFCC 54368.

*Notes*: This species is identified by having conidiomata with a column lenticular tissue in the center, and having multiple locules surrounded by a black conceptacle. It can be distinguished from its closest relative *C. coryli* by the discoid pycnidial stromata with a conspicuous central column, and smaller conidia (4.5–5.5 × 1–1.5 vs. 5–7 × 1–2 μm) (Zhu et al., 2020).

**Figure 4:**
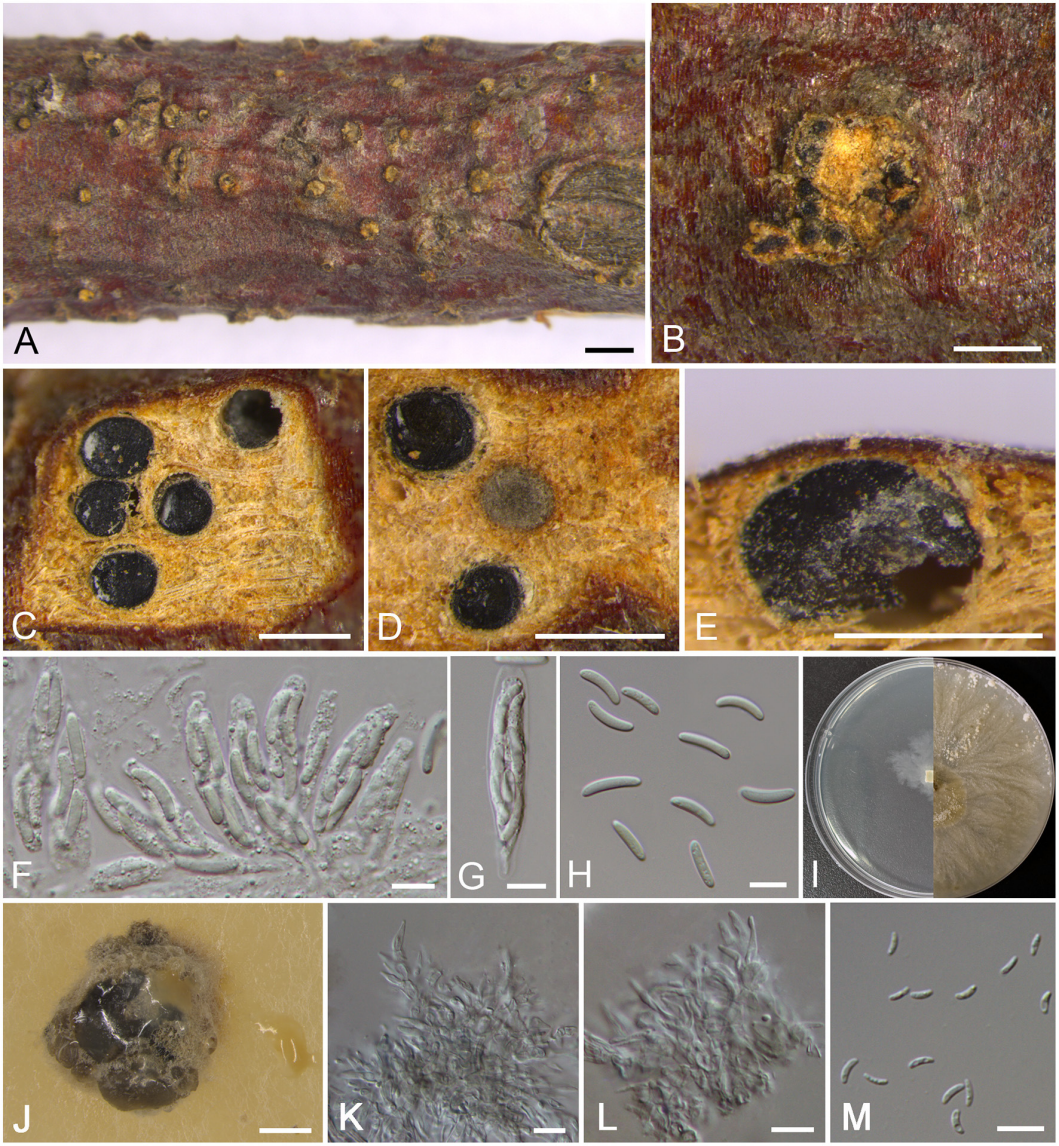
Morphology of *Cytospora donglingensis* (BJFC CF2019884). **(A,B)** Habit of ascomata on twig. **(C,D)** Transverse section of ascomata. **(E)** Longitudinal section through ascomata. **(F)** Asci and ascospores. **(G)** Ascus. **(H)** Ascospores. **(I)** Colonies on PDA at 3 days (left) and 30 days (right). **(J)** Conidiomata from culture on PDA. **(K,L)** Conidiophores. **(M)** Conidia. Bars: **(A)** = 1 mm; **(B–E), (J)** = 500 μm; **(F–H), (K–M)** = 10 μm.

***Cytospora donglingensis*** M. Pan & X.L. Fan, sp. nov. Figure 4

MycoBank MB 837634

*Typification*: CHINA. Beijing City: Mentougou District, Mount Dongling, Xiaolongmen Forestry Centre, 115°26′47.36″E, 39°56′06.45″N, from branches of *Platycladus orientalis*, 17 August 2017, *H.Y. Zhu* & *X.L. Fan* (**holotype** BJFC CF2019884, **isotype** BJM 240518), ex-type living culture CFCC 53159.

*Etymology*: Named after the location where it was collected, Mount Dongling.

*Descriptions*: Sexual morph: Ascostromata immersed in the bark, erumpent through the bark surface in a large area, scattered, with 5–8 perithecia arranged irregularly. Conceptacle absent. Ectostromatic disc buff, usually surrounded by ostiolar necks, triangular to circular, 550–900 μm in diam., with 4–10 ostioles arranged irregularly. Ostioles numerous, gray to black when mature, at the same or above level as the disc, arranged irregularly in a disc, 50–110 μm in diam. Perithecia gray to black when mature, flask-shaped to spherical, arranged irrugularly, 310–380 μm in diam. Asci hyaline, clavate to elongate-obovoid, 37.5–46.5 × 8–9.5 μm, 8-spored. Ascospores hyaline, elongate-allantoid, thin-walled, aseptate, 11–17 × 2.5–4 (av. = 13.8 ± 1.4 × 3 ± 0.4, *n* = 30) μm. Asexual morph: On PDA, pycnidial stromata covered by yellowish aerial mycelium, globose, solitary or aggregated deeply embedded in the medium, erumpent, black, 1,340–1,700 μm in diam., yellowish translucent to cream conidial drops exuding from the ostioles. Conidiophores coniform with the top end acute, hyaline, branched, 13–19 × 1.5–3.5 μm. Conidiogenous cells enteroblastic, phialidic. Conidia hyaline, allantoid, occasionally with little curve, rough, aseptate, unconspicuous biguttulate, 4.5–6 × 1–2 (av. = 5.4 ± 0.5 × 1.5 ± 0.2, *n* = 30) μm.

*Culture characteristics*: Cultures on PDA are initially white, growing slowly up to 2 cm in diam. after 3 days and entirely covering the 9 cm Petri dish after 7 days, becoming straw after 7–10 days. Colonies are flat with a uniform texture, producing pycnidia covered by sparse aerial mycelium with cream to yellowish conidial drops exuding from the ostioles after 30 days. Pycnidia aggregated on surface.

*Additional materials examined*: CHINA. Beijing City: Mentougou District, Mount Dongling, Xiaolongmen Forestry Centre, 115°26′47.36″E, 39°56′06.45″N, from branches of *Platycladus orientalis*, 17 August 2017, *H.Y. Zhu* & *X.L. Fan* (BJFC CF20201084), living culture CFCC 54371. Beijing City: Mentougou District, Mount Dongling, Xiaolongmen Forestry Centre, 115°23′39.32″E, 39°57′13.43″N, from branches of *Platycladus orientalis*, 17 August 2017, *H.Y. Zhu* & *X.L. Fan* (BJFC CF2019885), living culture CFCC 53160; *ibid*. BJFC CF20201085, living culture CFCC 54372.

*Notes*: In the phylogram, our new four isolates grouped in a separate clade with high statistical support (MP/ML/BI = 94/99/1) (Figure 1). Morphologically, *C. donglingensis* differs from other *Cytospora* species from *Platycladus orientalis* by the ascostroma without conceptacle, producing allantoid ascospores (11–17 × 2.5–4 μm). Thus, *C. donglingensis* is considered to represent a new species from *Platycladus orientalis*.

**Figure 5:**
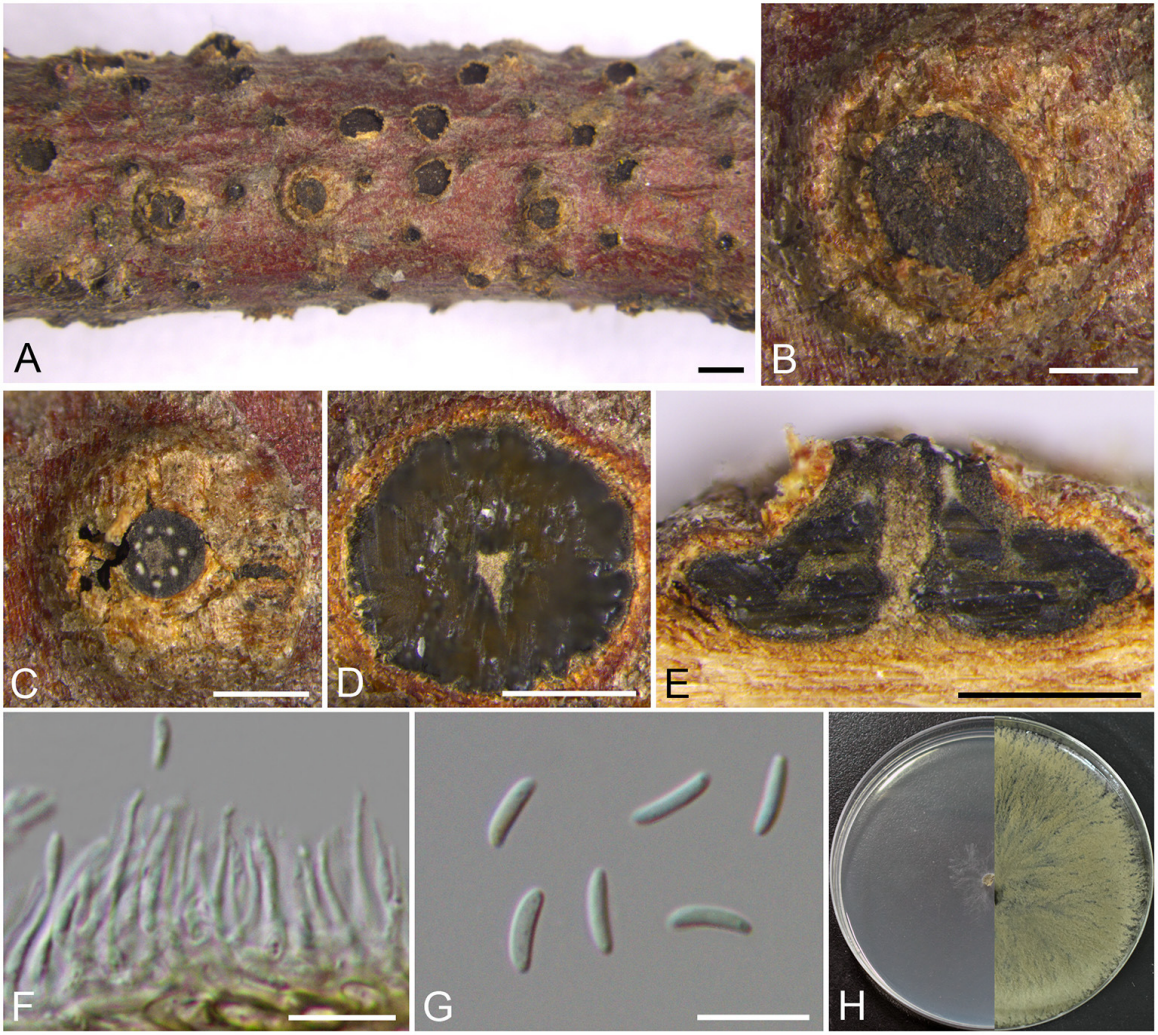
Morphology of *Cytospora verrucosa* (BJFC CF2019882). **(A–C)** Habit of conidiomata on twig. **(D)** Transverse section of conidioma. **(E)** Longitudinal section through conidioma. **(F)** Conidiophores and conidiogenous cells. **(G)** Conidia. **(H)** Colonies on PDA at 3 days (left) and 30 days (right). Bars: **(A)** = 1 mm; **(B–E)** = 500 μm; **(F,G)** = 10 μm.

***Cytospora verrucosa*** M. Pan & X.L. Fan, sp. nov. Figure 5

MycoBank MB 837635

*Typification*: CHINA. Beijing City: Mentougou District, Mount Dongling, Xiaolongmen Forestry Centre, 115°26′38.25″E, 39°57′18.47″N, from branches of *Platycladus orientalis*, 17 August 2017, *H.Y. Zhu* & *X.L. Fan* (**holotype** BJFC CF2019882, **isotype** BJM 240519), ex-type living culture CFCC 53157.

*Etymology*: Named after the verrucosa symptoms in branches.

*Descriptions*: Sexual morph: not observed. Asexual morph: Pycnidial stromata immersed in the bark, scattered erumpent through the surface of bark in a large area, verrucosa, with multiple locules and conspicuous central column. Central column beneath the disc more or less cylindrical, ochreous. Conceptacle absent. Ectostromatic disc brown to black, circular to ovoid, erumpent through the surface of bark in a large area, unconspicuous when mature, 580–940 μm in diam., with 5–7 ostioles per disc. Ostioles brown to black, arranged annularly on the disc, at the same or slightly above the level as the disc surface, 30–40 μm in diam. Locules numerous, subdivided frequently by invaginations with common walls, 990–1,190 μm in diam. Conidiophores approximately cylindrical with the top end acute, hyaline, unbranched, 10–13 × 0.5–1.5 μm. Conidiogenous cells enteroblastic, phialidic. Conidia hyaline, allantoid, smooth, aseptate, 6.5–8 × 1.5–2 (av. = 7.3 ± 0.4 × 1.7 ± 0.1, *n* = 30) μm.

*Culture characteristics*: Cultures on PDA are initially white, growing up to 6 cm in diam. after 3 days and entirely covering the 9 cm Petri dish after 5 days, becoming buff to honey after 7–10 days. Colonies are flat with a uniform texture; sterile.

*Additional materials examined*: CHINA. Beijing City: Mentougou District, Mount Dongling, Xiaolongmen Forestry Centre, 115°26′38.25″E, 39°57′18.47″N, from branches of *Platycladus orientalis*, 17 August 2017, *H.Y. Zhu* & *X.L. Fan* (BJFC CF20201082), living culture CFCC 54369. Beijing City: Mentougou District, Mount Dongling, Xiaolongmen Forestry Centre, 115°27′23.07″E, 39°58′26.37″N, from branches of *Platycladus orientalis*, 17 August 2017, *H.Y. Zhu* & *X.L. Fan* (BJFC CF2019883), living culture CFCC 53158; *ibid*. BJFC CF20201083, living culture CFCC 54370.

*Notes*: Phylogenetically, our new four isolates cluster in a separate lineage (MP/ML/BI = 100/100/1) comparing to other strains included in this study (Figure 1). Morphologically, *C. verrucosa* has similar characteristics to *C. friesii*, but it can be identified by having verrucosa symptoms in branches and pycnidia with a central column, and having numerous ostioles on a large area of the ectostromatic disc. Moreover, *C. verrucosa* differs from the closest species *C. globosa* by larger size of conidia (6.5–8 × 1.5–2 vs. 4–6.5 × 1–2 μm) (Li et al., 2020).

### Other Species Recorded From Coniferous Trees in China

***Cytospora beilinensis*** X.L. Fan & C.M. Tian, Persoonia, 45: 14, 2020.

*Notes*: *Cytospora beilinensis* has been reported from twigs and branches of *Pinus armandii* by Fan et al. (2020) in China, which has the same host with *C. pini* (Saccardo, 1884). Morphologically, *C. beilinensis* has larger conidia compared to *C. pini* (6–6.5 × 1–1.5 vs. 4 × 1 μm) (Saccardo, 1884; Fan et al., 2020).

***Cytospora bungeanae*** X.L. Fan & C.M. Tian, Persoonia, 45: 15, 2020.

*Notes*: In this study, *Cytospora beilinensis, C. bungeanae*, and *C. pini* were associated with *Pinus* spp. *Cytospora bungeanae* is characterized by conidiomata with black and inconspicuous ostiole and numerous locules which arranged irregularly with individual walls (Fan et al., 2020). *Cytospora bungeanae* differs from *C. beilinensis* and *C. pini* by the conidia size (4–5 × 1 vs. 6–6.5 × 1–1.5 μm, 4 × 1 μm) (Saccardo, 1884; Fan et al., 2020).

***Cytospora gigaspora*** C.M. Tian, X.L. Fan & K.D. Hyde, Phytotaxa, 197: 232, 2015.

*Notes*: *Cytospora gigalocus* was recorded from *Salix psammophila* and *Juniperus procumbens* (Fan et al., 2015b, 2020), which was similar with *C. nivea* regarded as the pathogen for poplar and willow canker (Saccardo, 1884; Teng, 1963; Tai, 1979; Wei, 1979; Zhuang, 2005; Fan et al., 2014b). However, *C. gigalocus* differs from other *Cytospora* species by the flat locules and larger conidia size (10.4 × 2.2 μm) (Fan et al., 2015b).

***Cytospora juniperina*** X.L. Fan & C.M. Tian, Persoonia, 45: 27, 2020.

*Notes*: *Cytospora juniperina* was described by Fan et al. (2020) associated with canker disease of *Juniperus przewalskii* in China. It is characterized by 5–12 perithecia arranged circularly or irregularly producing biseriate, elongate-allantoid, hyaline, aseptate ascospores (10–13.5 × 3–3.5 μm), and pycnidia with multiple locules producing allantoid, hyaline, aseptate conidia (6–6.5 × 1–1.5 μm) (Fan et al., 2020). Moreover, it has a unique characteristic owning a prominent white ectostromatic disc in symptomatic branches to easily diagnose.

***Cytospora piceae*** Fan, Phytotaxa, 383: 188, 2018.

*Notes*: *Cytospora piceae* was described by Pan et al. (2018) associated with canker disease of *Picea crassifolia* in Xinjiang, China. Phylogenetically, *C. piceae* is closed to *C. verrucosa* and *C. globosa*. It can be distinguished from *C. verrucosa* by the absent of central column and larger conidia (5–5.5 × 1–1.5 vs. 6.5–8 × 1.5–2 μm) (Pan et al., 2018). *Cytospora piceae* has a similar size of conidia with *C. globosa*, but *C. piceae* differs from *C. globosa* by larger conidiomata (720–1,190 vs. 400–550 μm) and smaller ostiole (70–115 vs. 100–220 μm) (Pan et al., 2018; Li et al., 2020).

***Cytospora platycladi*** X.L. Fan & C.M. Tian, Persoonia, 45: 33, 2020.

*Notes*: *Cytospora platycladi* was isolated from infected branches or twigs of *Platycladus orientalis*. Phylogenetically, *C. platycladi* formed a close group with *C. lumnitzericola* and *C. pingbianensis*. It differs from *C. lumnitzericola* by its disease symptoms with buff colored bark and the size of its conidia (4.5–5 × 1–1.5 vs. 4–5.5–1–1.3 μm) (Norphanphoun et al., 2018). It can also be distinguished from *C. pingbianensis* which is only introduced as a sexual morph by the phylogenetic position (Shang et al., 2020).

***Cytospora platycladicola*** X.L. Fan & C.M. Tian, Persoonia, 45: 33, 2020.

*Notes*: *Cytospora platycladicola* is associated with canker disease of *Platycladus orientalis* in China, which has same host with *C. platycladi*. *Cytospora platycladicola* can be distinguished from *C. platycladi* by the common walls of its locules (Fan et al., 2020).

**Key to** ***Cytospora* species on** ***Platycladus* spp. in China**

1 Sexual morph present.............. 2
1 Sexual morph absent............. 4
2 Ascostromata without conceptacle......... 3
2 Ascostromata with conceptacle.......... *C. albodisca*
3 Size of asci more than 47 μm........... *C. platycladicola*
3 Size of asci less than 47 μm.......... *C. donglingsis*
4 Pycnidium without conceptacle.......... 5
4 Pycnidium with conceptacle.......... *C. discostoma*
5 Pycnidial stromata with single ostiole............ *C. platycladi*
5 Pycnidial stromata with numerous ostioles............ *C. verrucosa*

**Key to *Cytospora* species on coniferous trees in China**

1 Sexual morph present.............. 2
1 Sexual morph absent............ 5
2 Ascostromata without conceptacle............ 3
2 Ascostromata with conceptacle.............. *C. albodisca*
3 Size of asci more than 47 μm...... *C. platycladicola*
3 Size of asci less than 47 μm ......... 4
4 Size of conidia more than 5.5 μm......... *C. juniperina*
4 Size of conidia less than 5.5 μm........ *C. donglingensis*
5 Pycnidium without conceptacle......... 6
5 Pycnidium with conceptacle.......... 10
6 Pycnidial stromata with single ostiole........... 7
6 Pycnidial stromata with numerous ostioles........... *C. verrucosa*
7 Locules with the common walls........... 8
7 Locules with the independent walls.............. *C. platycladi*
8 Host *Pinus* spp. .......... 9
8 Host *Picea* spp. ............ *C. piceae*
9 Size of conidia more than 5.5 μm.......... *C. bungeanae*
9 Size of conidia less than 5.5 μm.......... *C. beilinensis*
10 Pycnidial stromata without a central column... *C. gigaspora*
10 Pycnidial stromata with a central column... *C. discostoma*

## Discussion

In the present study, we utilized a polyphasic approach of molecular phylogenetic analyses of the combined alignment of ITS, LSU, *act, rpb2, tef1-*α, and *tub2* gene suquences along with morphological observations. Eleven *Cytospora* species represented by 28 strains from coniferous trees, including four new species (*C. albodisca, C. discostoma, C. donglingensis*, and *C. verrucosa*), and seven known species (*C. beilinensis, C. bungeanae, C. gigaspora, C. juniperina, C. piceae, C. platycladi*, and *C. platycladicola*) were evaluated. A dubious species, *C. curreyi*, was reported from *Abies* sp. in the Sichuan Province, China (Teng, 1963), but it was not confirmed due to the non-availability of living culture.

Consistent with the conclusions of a previous study, sexual morphs of *Cytospora* species associated with coniferous trees summarized herein are rarely found in nature. In the present study, among the specimens of the four new species, two new species (*C. albodisca* and *C. donglingensis*) had only sexual morphs, and the other two new species (*C. discostoma* and *C. verrucosa*) had only asexual morphs. Therefore, we observed the asexual morphs of *C. albodisca* and *C. donglingensis* after sporulation on PDA medium. Adams et al. (2005) reported that the asexual morphs of *Cytospora* formed naturally may be different from those formed in culture, and these morphological characteristics may not be meaningful in classification. In conidia morphology, *C. discostoma* resembles *C. donglingensis* (4.5–5.5 × 1–1.5 vs. 4.5–6 × 1–2 μm). However, *C. discostoma* differs from *C. donglingensis* in ITS (33/656), LSU (7/522), *act* (61/367), *rpb2* (71/726), *tef1-*α (104/824), and *tub2* (101/648). We found that the size of the conidia was distinguishable from that of other species, and this was strongly supported by DNA sequence data.

Host affiliation has been primarily used as the delimitation in *Cytospora* in the early stage, and this has been proven uninformative because several *Cytospora* species have been discovered in a wide range of hosts (Adams et al., 2005, 2006; Lawrence et al., 2018; Norphanphoun et al., 2018; Fan et al., 2020; Pan et al., 2020). Lawrence et al. (2018) reported that *Cytospora* included generalist and specialist pathogens by taking *C. chrysosperma* and *C. punicae* as examples; however, a clear elucidation of the host ranges and distribution of *Cytospora* species will require a more exhaustive sampling of other coniferous trees from other regions of the world. Consistent with previous findings, some *Cytospora* species isolated from coniferous trees occurred on different hosts (i.e., *C. ampulliformis, C. gigaspora, C. melnikii*) (Fan et al., 2015b, 2020; Norphanphoun et al., 2017, 2018; Lawrence et al., 2018) rather than specific hosts. In addition, it cannot be denied that some species of *Cytospora* have a preference for certain hosts (i.e., *C. japonica* with chiefly Rosaceae host record, *C. mali* with apple host record and *C. pini* with pine host record) (Teng, 1963; Tai, 1979; Wei, 1979; Zhuang, 2005; Wang et al., 2011). In our study, all species with available strains found in China were associated with a single coniferous host (mainly *Juniperus, Picea, Pinus*, and *Platycladus*), with the exception of *Cytospora gigaspora*, which has also been reported in *Salix psammophila* (Fan et al., 2015b). These findings suggest that future studies are needed to better understand the interaction between fungi and their hosts.

Coniferous trees are the main timber and greening tree species in forestry production, but they are exposed to different pathogens. *Cytospora* was recorded on the hosts of four coniferous families (14 *Cytospora* spp. infecting Cupressaceae, 14 *Cytospora* spp. infecting Pinaceae, three *Cytospora* spp. infecting Taxaceae and three *Cytospora* spp. infecting Taxodiaceae) based on the U.S. National Fungus Collections Fungus-Host database (Farr and Rossman, 2021), but most species are inadequately identified and lack of molecular data. In China, some new species and new records of *Cytospora* on conifers have been reported successively (Fan et al., 2015b, 2020; Pan et al., 2018), lacking a systematic study summarizing *Cytospora* species isolated from conifers. The present study indicated that the common families of conifers infected by *Cytospora* are Cupressaceae and Pinaceae, although several coniferous hosts suffering from canker disease have not been discovered. Thus, the present research is preliminary in nature, and further studies using a more intensive and wider sampling of isolates are awaited.

*Cytospora* canker and dieback diseases present with different symptoms in hardwoods and conifers. In hardwoods, the symptoms are characterized by elongated, slightly sunken, and discolored areas in the bark with obvious black spots (Fan et al., 2020). However, discoloration of the adjacent cambium in conifers has not been observed, although the fungus can be isolated from nearby xylem (Schoeneweiss, 1983). A large amount of resin flows from the infected branches, covers the bark surface surrounded by the canker, and drips onto the lower branches (Schoeneweiss, 1983). Generally, in canker disease, *Cytospora* begins to infect through cracks and wounds in the bark. The wounds include pruning wounds, cold injuries, leaf scars, and branches with weakened shade. If perennial cankers originating from pruning wounds occur in places critical to the strength of the trees, they can be highly destructive (Biggs, 1989; Adams et al., 2006). Therefore, the occurrence of *Cytospora* canker and dieback diseases can be minimized by maintaining susceptible trees as strong as possible, and by removing dead and dying branches in the dry season. All unnecessary damage should be avoided. Moreover, the occurrence of *Cytospora* canker diseases is not only affected by the environment and distribution, but also by transmission (Fan et al., 2015b), which may act as potential inoculum sources for other hosts in natural and artificial environments. Six pathogens, including *Cytospora*, have been found to pose a high risk of causing severe damage if exported to other suitable environments (Cannon et al., 2016). At present, the host specificity and pathogenicity of several *Cytospora* species associated with coniferous trees are poorly known. In the subsequent studies, more attention should be paid to the pathogenicity and aggressiveness of *Cytospora*, which can play a role in quarantine, monitoring, and early warning of forestry pathogen.

In conclusion, in this study, we focused on four *Cytospora* species isolated from *Platycladus orientalis* in China. Our study implies that many additional *Cytospora* species from China are still undiscovered. The keys to *Cytospora* species on *Platycladus* spp. and coniferous trees were established based on their unique morphological characteristics, and this will also help more extensive research on fungal pathogens in China. Furthermore, this study constitutes a step toward the taxonomic study of conifer pathogens, providing sustainable disease management strategies for conifers infected by *Cytospora* species in China.

## Data Availability Statement

The datasets presented in this study can be found in online repositories. The names of the repository/repositories and accession number(s) can be found in the article/Supplementary Material.

## Author Contributions

XF and CT: conceived and designed the experiments. MP, HZ, and MH: performed the experiments. MP and HZ: analyzed the data. MP: wrote the manuscript. XF: revised and approved the final version of the paper. All authors contributed extensively to the work presented in this paper.

## Conflict of Interest

The authors declare that the research was conducted in the absence of any commercial or financial relationships that could be construed as a potential conflict of interest.
