# Supplementary material for: Assessment of Cytospora Isolates From Conifer Cankers in China, With the Descriptions of Four New Cytospora Species
Source: Front Plant Sci. 2021 Feb 18;12:636460. doi: 10.3389/fpls.2021.636460 (PMC7930227; doi:10.3389/fpls.2021.636460)
Supplement: Supplementary file 1 [file Table_1.DOCX]

**Table S1.** Strains of *Cytospora* used in the molecular analyses in this study.

| Species | Strain1 | Host | Origin | GenBank accession numbers | | | | | |
| --- | --- | --- | --- | --- | --- | --- | --- | --- | --- |
|  |  |  |  | ITS | LSU | *act1* | *rpb2* | *tef1-α* | *tub2* |
| *C. ailanthicola* | CFCC 89970T | *Ailanthus altissima* | Ningxia, China | MH933618 | MH933653 | MH933526 | MH933592 | MH933494 | MH933565 |
| *C. abyssinica* | CMW 10181T | *Eucalyptus globulus* | Ethiopia | AY347353 | NA | NA | NA | NA | NA |
| *C. abyssinica* | CMW 10178 | *Eucalyptus globulus* | Ethiopia | AY347354 | NA | NA | NA | NA | NA |
| *C. abyssinica* | CMW 10179 | *Eucalyptus globulus* | Ethiopia | AY347352 | NA | NA | NA | NA | NA |
| *C. acaciae* | CBS 468.69 | *Ceratonia siliqua* | Spain | DQ243804 | NA | NA | NA | NA | NA |
| ***C. albodisca*** | **CFCC 53161T*** | *Platycladus orientalis* | Beijing, China | MW418406 | MW418418 | MW422899 | MW422909 | MW422921 | MW422933 |
| ***C. albodisca*** | **CFCC 54373*** | *Platycladus orientalis* | Beijing, China | MW418407 | MW418419 | MW422900 | MW422910 | MW422922 | MW422934 |
| *C. ampulliformis* | MFLUCC 16-0583T | *Sorbus intermedia* | Russia | KY417726 | KY417760 | KY417692 | KY417794 | NA | NA |
| *C. ampulliformis* | MFLUCC 16-0629 | *Acer platanoides* | Russia | KY417727 | KY417761 | KY417693 | KY417795 | NA | NA |
| *C. amygdali* | CBS 144233T | *Prunus dulcis* | California, USA | MG971853 | NA | MG972002 | NA | MG971659 | MG971718 |
| *C. atrocirrhata* | CFCC 89615 | *Juglans regia* | Qinghai, China | KR045618 | KR045700 | KF498673 | KU710946 | KP310858 | KR045659 |
| *C. atrocirrhata* | CFCC 89616 | *Juglans regia* | Qinghai, China | KR045619 | KR045701 | KF498674 | KU710947 | KP310859 | KR045660 |
| *C. atrocirrhata* | 22 | *Salix excelsa* | Iran | EF447305 | NA | NA | NA | NA | NA |
| *C. austromontana* | CMW 6735T | *Eucalyptus pauciflora* | Australia | AY347361 | NA | NA | NA | NA | NA |
| *C. beilinensis* | CFCC 50493T* | *Pinus armandii* | Beijing, China | MH933619 | MH933654 | MH933527 | NA | MH933495 | MH933561 |
| *C. beilinensis* | CFCC 50494* | *Pinus armandii* | Beijing, China | MH933620 | MH933655 | MH933528 | NA | MH933496 | MH933562 |
| *C. berberidis* | CFCC 89927T | *Berberis dasystachya* | Qinghai, China | KR045620 | KR045702 | KU710990 | KU710948 | KU710913 | KR045661 |
| *C. berberidis* | CFCC 89933 | *Berberis dasystachya* | Qinghai, China | KR045621 | KR045703 | KU710991 | KU710949 | KU710914 | KR045662 |
| *C. berkeleyi* | StanfordT3T | *Eucalyptus globulus* | USA | AY347350 | NA | NA | NA | NA | NA |
| *C. berkeleyi* | UCBTwig3 | *Eucalyptus globulus* | USA | AY347349 | NA | NA | NA | NA | NA |
| *C. brevispora* | CBS 116829 | *Eucalyptus grandis* | Venezuela | AF192321 | NA | NA | NA | NA | NA |
| *C. brevispora* | CBS 116811T | *Eucalyptus grandis × tereticornis* | Congo | AF192315 | NA | NA | NA | NA | NA |
| *C. bungeana* | CFCC 50495T* | *Pinus bungeana* | Shanxi, China | MH933621 | MH933656 | MH933529 | MH933593 | MH933497 | MH933563 |
| *C. bungeana* | CFCC 50496* | *Pinus bungeana* | Shanxi, China | MH933622 | MH933657 | MH933530 | MH933594 | MH933498 | MH933564 |
| *C. californica* | CBS 144234T | *Juglans regia* | California, USA | MG971935 | NA | MG972083 | NA | MG971645 | NA |
| *C. castanae* | DBT 183T | *Castanea sativa* | North India | KC963921 | NA | NA | NA | NA | NA |
| *C. carbonacea* | CFCC 89947 | *Ulmus pumila* | Qinghai, China | KR045622 | KP310812 | KP310842 | KU710950 | KP310855 | KP310825 |
| *C. carpobroti* | CMW 48981T | *Carpobrotus edulis* | South Africa | MH382812 | MH411216 | NA | NA | MH411212 | MH411207 |
| *C. cedri* | CBS 196.50 | NA | Italy | AF192311 | NA | NA | NA | NA | NA |
| *C. celtidicola* | CFCC 50497T | *Celtis sinensis* | Anhui, China | MH933623 | MH933658 | MH933531 | MH933595 | MH933499 | MH933566 |
| *C. celtidicola* | CFCC 50498 | *Celtis sinensis* | Anhui, China | MH933624 | MH933659 | MH933532 | MH933596 | MH933500 | MH933567 |
| *C. centrivillosa* | MFLUCC 16-1206T | *Sorbus domestica* | Italy | MF190122 | MF190068 | NA | MF377600 | NA | NA |
| *C. centrivillosa* | MFLUCC 17-1660 | *Sorbus domestica* | Italy | MF190123 | MF190069 | NA | MF377601 | NA | NA |
| *C. ceratosperma* | CBS 116.21 | *Fagus sylvatica* | Netherlands | AY347335 | NA | NA | NA | NA | NA |
| *C. ceratosperma* | CBS 192.42 | *Taxus baccata* | Switzerland | AY347333 | NA | NA | NA | NA | NA |
| *C. ceratosperma* | CFCC 89624 | *Juglans regia* | Gansu, China | KR045645 | KR045724 | NA | KU710976 | KP310860 | KR045686 |
| *C. ceratosperma* | CFCC 89625 | *Juglans regia* | Gansu, China | KR045646 | KR045725 | NA | KU710977 | KP31086 | KR045687 |
| *C. ceratospermopsis* | CFCC 89626T | *Juglans regia* | Shaanxi, China | KR045647 | KR045726 | KU711011 | KU710978 | KU710934 | KR045688 |
| *C. ceratospermopsis* | CFCC 89627 | *Juglans regia* | Shaanxi, China | KR045648 | KR045727 | KU711012 | KU710979 | KU710935 | KR045689 |
| *C. chrysosperma* | CFCC 89629 | *Salix psammophila* | Shaanxi, China | KF765673 | KF765689 | NA | KF765705 | NA | NA |
| *C. chrysosperma* | CFCC 89981 | Populus alba subsp. pyramidalis | Gansu, China | MH933625 | MH933660 | MH933533 | MH933597 | MH933501 | MH933568 |
| *C. chrysosperma* | CFCC 89982 | *Ulmus pumila* | Tibet, China | KP281261 | KP310805 | KP310835 | NA | KP310848 | KP310818 |
| *C. cinerostroma* | CMW 5700T | *Eucalyptus globulus* | Chile | AY347377 | NA | NA | NA | NA | NA |
| *C. cinnamomea* | CFCC 53178T | *Prunus armeniaca* | Xinjiang, China | MK673054 | MK673084 | MK673024 | NA | NA | MK672970 |
| *C. coryli* | CFCC 53162T | *Corylus mandshurica* | Beijing, China | MN854450 | MN854661 | NA | MN850751 | MN850758 | MN861120 |
| *C. cotoneastricola* | CF 20197027 | Cotoneaster sp. | Tibet, China | MK673072 | MK673102 | MK673042 | MK673012 | MK672958 | MK672988 |
| *C. cotoneastricola* | CF 20197028 | Cotoneaster sp. | Tibet, China | MK673073 | MK673103 | MK673043 | MK673013 | MK672959 | MK672989 |
| *C. cotoneastricola* | CF 20197030 | Cotoneaster sp. | Tibet, China | MK673074 | MK673104 | MK673044 | MK673014 | MK672960 | MK672990 |
| *C. cotoneastricola* | CF 20197031T | Cotoneaster sp. | Tibet, China | MK673075 | MK673105 | MK673045 | MK673015 | MK672961 | MK672991 |
| *C. cotini* | MFLUCC 14-1050T | *Cotinus coggygria* | Russia | KX430142 | KX430143 | NA | KX430144 | NA | NA |
| *C. curvata* | MFLUCC 15-0865T | *Salix alba* | Russia | KY417728 | KY417762 | KY417694 | KY417796 | NA | NA |
| *C. davidiana* | CXY 1350T | *Populus davidiana* | Inner Mongolia, China | KM034870 | NA | NA | NA | NA | NA |
| *C. davidiana* | CXY 1374 | *Populus davidiana* | Heilongjiang, China | KM034869 | NA | NA | NA | NA | NA |
| *C. diatrypelloidea* | CMW 8549T | *Eucalyptus globulus* | Australia | AY347368 | NA | NA | NA | NA | NA |
| *C. diopuiensis* | MFLUCC 18-1419T | Undefined wood | Chiang Mai, Thailand | MK912137 | MK571765 | MN685819 | NA | NA | NA |
| *C. disciformis* | CMW 6509T | *Eucalyptus grandis* | Uruguay | AY347374 | NA | NA | NA | NA | NA |
| *C. disciformis* | CMW 6750 | *Eucalyptus globulus* | Australia | AY347359 | NA | NA | NA | NA | NA |
| ***C. discostoma*** | **CFCC 53137T*** | *Platycladus orientalis* | Beijing, China | MW418404 | MW418416 | MW422897 | MW422907 | MW422919 | MW422931 |
| ***C. discostoma*** | **CFCC 54368*** | *Platycladus orientalis* | Beijing, China | MW418405 | MW418417 | MW422898 | MW422908 | MW422920 | MW422932 |
| ***C. donglingensis*** | **CFCC 53159T*** | *Platycladus orientalis* | Beijing, China | MW418412 | MW418424 | MW422903 | MW422915 | MW422927 | MW422939 |
| ***C. donglingensis*** | **CFCC 53160*** | *Platycladus orientalis* | Beijing, China | MW418414 | MW418426 | MW422905 | MW422917 | MW422929 | MW422941 |
| ***C. donglingensis*** | **CFCC 54371*** | *Platycladus orientalis* | Beijing, China | MW418413 | MW418425 | MW422904 | MW422916 | MW422928 | MW422940 |
| ***C. donglingensis*** | **CFCC 54372*** | *Platycladus orientalis* | Beijing, China | MW418415 | MW418427 | MW422906 | MW422918 | MW422930 | MW422942 |
| *C. elaeagni* | CFCC 89632 | *Elaeagnus angustifolia* | Ningxia, China | KR045626 | KR045706 | KU710995 | KU710955 | KU710918 | KR045667 |
| *C. elaeagni* | CFCC 89633 | *Elaeagnus angustifolia* | Ningxia, China | KF765677 | KF765693 | KU710996 | KU710956 | KU710919 | KR045668 |
| *C. elaeagnicola* | CFCC 52882T | *Elaeagnus angustifolia* | China | MK732341 | MK732338 | MK732344 | MK732347 | NA | NA |
| *C. elaeagnicola* | CFCC 52883 | *Elaeagnus angustifolia* | China | MK732342 | MK732339 | MK732345 | MK732348 | NA | NA |
| *C. elaeagnicola* | CFCC 52884 | *Elaeagnus angustifolia* | China | MK732343 | MK732340 | MK732346 | MK732349 | NA | NA |
| *C. eriobotryae* | IMI 136523T | *Eriobotrya japonica* | India | AY347327 | NA | NA | NA | NA | NA |
| *C. erumpens* | CFCC 50022 | *Prunus padus* | Shanxi, China | MH933627 | MH933661 | MH933534 | NA | MH933502 | MH933569 |
| *C. erumpens* | MFLUCC 16-0580T | Salix × fragilis | Russia | KY417733 | KY417767 | KY417699 | KY417801 | NA | NA |
| *C. erumpens* | CFCC 53163 | *Prunus padus* | Xinjiang, China | MK673059 | MK673089 | MK673029 | MK673000 | MK672948 | MK672975 |
| *C. eucalypti* | LSEQ | *Sequoia sempervirens* | USA | AY347340 | NA | NA | NA | NA | NA |
| *C. eucalypti* | CBS 144241 | *Eucalyptus globulus* | California, USA | MG971907 | NA | MG972056 | NA | MG971617 | MG971772 |
| *C. eucalypticola* | ATCC 96150T | *Eucalyptus nitens* | Australia | AY347358 | NA | NA | NA | NA | NA |
| *C. eucalypticola* | CMW 5309 | *Eucalyptus grandis* | Uganda | AF260266 | NA | NA | NA | NA | NA |
| *C. eucalyptina* | CMW 5882 | *Eucalyptus grandis* | Columbia | AY347375 | NA | NA | NA | NA | NA |
| *C. eugeniae* | CMW 7029 | Tibouchina sp. | Australia | AY347364 | NA | NA | NA | NA | NA |
| *C. eugeniae* | CMW 8648 | *Eugenia* sp. | Indonesia | AY347344 | NA | NA | NA | NA | NA |
| *C. euonymicola* | CFCC 50499T | *Euonymus kiautschovicus* | Shaanxi, China | MH933628 | MH933662 | MH933535 | MH933598 | MH933503 | MH933570 |
| *C. euonymicola* | CFCC 50500 | *Euonymus kiautschovicus* | Shaanxi, China | MH933629 | MH933663 | MH933536 | MH933599 | MH933504 | MH933571 |
| *C. euonymina* | CFCC 89993T | *Euonymus kiautschovicus* | Shanxi, China | MH933630 | MH933664 | MH933537 | MH933600 | MH933505 | MH933590 |
| *C. euonymina* | CFCC 89999 | *Euonymus kiautschovicus* | Shanxi, China | MH933631 | MH933665 | MH933538 | MH933601 | MH933506 | MH933591 |
| *C. fraxiicola* | MFLU 17-2392 | dead branches | Russia | NA | MN764356 | MN995562 | NA | NA | NA |
| *C. fraxinigena* | MFLU 17-0880 | *Fraxinus ornus* | Italy | MF190134 | MF190079 | NA | NA | NA | NA |
| *C. fraxinigena* | MFLUCC 14-0868T | *Fraxinus ornus* | Italy | MF190133 | MF190078 | NA | NA | NA | NA |
| *C. friesii* | CBS 194.42 | *Abies alba* | Switzerland | AY347328 | NA | NA | NA | NA | NA |
| *C. fugax* | CXY 1371 | *Populus simonii* | Jilin, China | KM034852 | NA | NA | NA | NA | KM034891 |
| *C. fugax* | CXY 1381 | *Populus ussuriensis* | Heilongjiang, China | KM034853 | NA | NA | NA | NA | KM034890 |
| *C. fugax* | CBS 203.42 | Salix sp. | Switzerland | AY347323 | NA | NA | NA | NA | NA |
| 1. *fusispora* | AMH 10073^T^ | unidentifed decaying twig | India | MN227694 | MN250032 | NA | NA | NA | NA |
| *C. galegicola* | MFLUCC 18-1199T | *Galega officinalis* | Forlì-Cesena, Italy | MK912128 | MK571756 | MN685810 | MN685820 | NA | NA |
| *C. germanica* | CXY 1322 | *Elaeagnus oxycarpa* | China | JQ086563 | JX524617 | NA | NA | NA | NA |
| *C. gigalocus* | CFCC 89620T | *Juglans regia* | Qinghai, China | KR045628 | KR045708 | KU710997 | KU710957 | KU710920 | KR045669 |
| *C. gigalocus* | CFCC 89621 | *Juglans regia* | Qinghai, China | KR045629 | KR045709 | KU710998 | KU710958 | KU710921 | KR045670 |
| *C. gigaspora* | CFCC 50014* | *Juniperus procumbens* | Shanxi, China | KR045630 | KR045710 | KU710999. | KU710959 | KU710922 | KR045671 |
| *C. gigaspora* | CFCC 89634T* | *Salix psammophila* | Shaanxi, China | KF765671 | KF765687 | KU711000 | KU710960 | KU710923 | KR045672 |
| 1. *globosa* | MFLU 16-2054^T^ | *Abies alba* | Italy | MT177935 | MT177962 | NA | MT432212 | MT454016 | NA |
| *C. granati* | CBS 144237T | *Punica granatum* | California, USA | MG971799 | NA | MG971949 | NA | MG971514 | MG971664 |
| *C. hippophaës* | CFCC 89639 | *Hippophaë rhamnoides* | Gansu, China | KR045632 | KR045712 | KU711001 | KU710961 | KU710924 | KR045673 |
| *C. hippophaës* | CFCC 89640 | *Hippophaë rhamnoides* | Gansu, China | KF765682 | KF765698 | KF765730 | KU710962 | KP310865 | KR045674 |
| *C. japonica* | CBS 375.29 | *Prunus persica* | Japan | AF191185 | NA | NA | NA | NA | NA |
| *C. japonica* | CFCC 89956 | *Prunus cerasifera* | Ningxia, China | KR045624 | KR045704 | KU710993 | KU710953 | KU710916 | KR045665 |
| *C. japonica* | CFCC 89960 | *Prunus cerasifera* | Ningxia, China | KR045625 | KR045705 | KU710994 | KU710954 | KU710917 | KR045666 |
| *C. joaquinensis* | CBS 144235T | *Populus deltoides* | California, USA | MG971895 | NA | MG972044 | NA | MG971605 | MG971761 |
| *C. junipericola* | BBH 42444 | *Juniperus communis* | Italy | MF190126 | MF190071 | NA | NA | MF377579 | NA |
| *C. junipericola* | MFLU 17-0882T | *Juniperus communis* | Italy | MF190125 | MF190072 | NA | NA | MF377580 | NA |
| *C. juniperina* | CFCC 50501T* | *Juniperus przewalskii* | Sichuan, China | MH933632 | MH933666 | MH933539 | MH933602 | MH933507 | NA |
| *C. juniperina* | CFCC 50502* | *Juniperus przewalskii* | Sichuan, China | MH933633 | MH933667 | MH933540 | MH933603 | MH933508 | MH933572 |
| *C. juniperina* | CFCC 50503* | *Juniperus przewalskii* | Sichuan, China | MH933634 | MH933668 | MH933541 | MH933604 | MH933509 | NA |
| *C. kantschavelii* | CXY 1383 | *Populus maximowiczii* | Jilin, China | KM034867 | NA | NA | NA | NA | NA |
| *C. kantschavelii* | CXY 1386 | *Populus maximowiczii* | Chongqing, China | KM034867 | NA | NA | NA | NA | NA |
| *C. kuanchengensis* | CFCC 52464T | *Castanea mollissima* | China | MK432616 | MK429886 | MK442940 | MK578076 | NA | NA |
| *C. kuanchengensis* | CFCC 52465 | *Castanea mollissima* | China | MK432617 | MK429887 | MK442941 | MK578077 | NA | NA |
| *C. kunzei* | CBS 118556 | *Pinus radiata* | South Africa | DQ243791 | NA | NA | NA | NA | NA |
| *C. longiostiolata* | MFLUCC 16-0628T | Salix × fragilis | Russia | KY417734 | KY417768 | KY417700 | KY417802 | NA | NA |
| *C. longispora* | CBS 144236T | *Prunus domestica* | California, USA | MG971905 | NA | MG972054 | NA | MG971615 | MG971764 |
| *C. leucosperma* | CFCC 89622 | *Pyrus bretschneideri* | Gansu, China | KR045616 | KR045698 | KU710988 | KU710944 | KU710911 | KR045657 |
| *C. leucosperma* | CFCC 89894 | *Pyrus bretschneideri* | Qinghai, China | KR045617 | KR045699 | KU710989 | KU710945 | KU710912 | KR045658 |
| *C. leucostoma* | CFCC 50016 | *Sorbus aucuparia* | Ningxia, China | MH820400 | MH820393 | MH820408 | NA | MH820404 | MH820389 |
| *C. leucostoma* | CFCC 50017 | *Prunus cerasifera* | Ningxia, China | MH933635 | MH933669 | MH933542 | NA | MH933510 | MH933573 |
| *C. leucostoma* | CFCC 50018 | *Prunus serrulata* | Gansu, China | MH933636 | MH933670 | MH933543 | NA | MH933511 | MH933574 |
| *C. leucostoma* | CFCC 50019 | *Rosa helenae* | Gansu, China | MH933637 | MH933671 | MH933544 | NA | NA | NA |
| *C. leucostoma* | CFCC 50020 | *Prunus persica* | Gansu, China | MH933638 | MH933672 | MH933545 | NA | NA | NA |
| *C. leucostoma* | CFCC 50021 | *Prunus salicina* | Gansu, China | MH933639 | MH933673 | MH933546 | NA | MH933512 | MH933575 |
| *C. leucostoma* | CFCC 50023 | *Cornus alba* | Shanxi, China | KR045635 | KR045715 | KU711003 | KU710964 | KU710926 | KR045676 |
| *C. leucostoma* | CFCC 50024 | *Prunus pseudocerasus* | Qinghai, China | MH933640 | MH933674 | MH933547 | MH933605 | NA | MH933576 |
| *C. leucostoma* | CFCC 50467 | *Betula platyphylla* | Beijing, China | KT732948 | KT732967 | NA | NA | NA | NA |
| *C. leucostoma* | CFCC 50468 | *Betula platyphylla* | Beijing, China | KT732949 | KT732968 | NA | NA | NA | NA |
| *C. leucostoma* | CFCC 53140 | *Prunus sibirica* | Beijing, China | MN854445 | MN854656 | MN850760 | MN850746 | MN850753 | MN861115 |
| *C. leucostoma* | CFCC 53141 | *Prunus sibirica* | Beijing, China | MN854446 | MN854657 | MN850761 | MN850747 | MN850754 | MN861116 |
| *C. leucostoma* | CFCC 53156 | *Juglans mandshurica* | Beijing, China | MN854447 | MN854658 | MN850762 | MN850748 | MN850755 | MN861117 |
| *C. leucostoma* | MFLUCC 16-0574 | Rosa sp. | Russia | KY417731 | KY417764 | KY417696 | KY417798 | NA | NA |
| *C. leucostoma* | MFLUCC 16-0589 | *Salix alba* | Russia | KY417732 | KY417766 | KY417698 | KY417800 | NA | NA |
| *C. leucostoma* | CFCC 53165 | *Sorbus tianschanica* | Xinjiang, China | MK673053 | MK673083 | MK673023 | NA | MK672944 | MK672969 |
| *C. leucostoma* | CFCC 53166 | *Prunus armeniaca* | Xinjiang, China | MK673055 | MK673085 | MK673025 | NA | MK672945 | MK672971 |
| *C. leucostoma* | CFCC 53167 | *Prunus armeniaca* | Xinjiang, China | MK673056 | MK673086 | MK673026 | MK672998 | MK672946 | MK672972 |
| *C. leucostoma* | CFCC 53168 | *Prunus pseudocerasus* | Xinjiang, China | MK673063 | MK673093 | MK673033 | NA | NA | MK672979 |
| *C. leucostoma* | CFCC 53169 | *Prunus persica* | Beijing, China | MK673080 | MK673110 | MK673050 | MK673020 | MK672966 | MK672996 |
| *C. leucostoma* | CFCC 53170 | *Prunus persica* | Beijing, China | MK673081 | MK673111 | MK673051 | MK673021 | MK672967 | MK672997 |
| *C. lumnitzericola* | MFLUCC 17-0508T | *Lumnitzera racernosa* | Tailand | MG975778 | MH253461 | MH253457 | MH253453 | NA | NA |
| *C. mali* | CFCC 50028 | *Malus pumila* | Gansu, China | MH933641 | MH933675 | MH933548 | MH933606 | MH933513 | MH933577 |
| *C. mali* | CFCC 50029 | *Malus pumila* | Ningxia, China | MH933642 | MH933676 | MH933549 | MH933607 | MH933514 | MH933578 |
| *C. mali* | CFCC 50030 | *Malus pumila* | Shaanxi, China | MH933643 | MH933677 | MH933550 | MH933608 | MH933524 | MH933579 |
| *C. mali* | CFCC 50031 | Crataegus sp. | Shanxi, China | KR045636 | KR045716 | KU711004 | KU710965 | KU710927 | KR045677 |
| *C. mali* | CFCC 50044 | *Malus baccata* | Qinghai, China | KR045637 | KR045717 | KU711005 | KU710966 | KU710928 | KR045678 |
| *C. mali-spectabilis* | CFCC 53181T | *Malus spectabilis* ‘Royalty’ | Xinjiang, China | MK673066 | MK673096 | MK673036 | MK673006 | MK672953 | MK672982 |
| *C. melnikii* | CFCC 89984 | *Rhus typhina* | Xinjiang, China | MH933644 | MH933678 | MH933551 | MH933609 | MH933515 | MH933580 |
| *C. melnikii* | MFLUCC 15-0851T | *Malus domestica* | Russia | KY417735 | KY417769 | KY417701 | KY417803 | NA | NA |
| *C. melnikii* | MFLUCC 16-0635 | Populus nigra var. italica | Russia | KY417736 | KY417770 | KY417702 | KY417804 | NA | NA |
| *C. mougeotii* | ATCC 44994 | *Picea abies* | Norway | AY347329 | NA | NA | NA | NA | NA |
| *C. multicollis* | CBS 105.89T | Quercus ilex subsp. rotundifolia | Spain | DQ243803 | NA | NA | NA | NA | NA |
| *C. myrtagena* | CFCC 52454 | *Castanea mollissima* | China | MK432614 | MK429884 | MK442938 | MK578074 | NA | NA |
| *C. myrtagena* | CFCC 52455 | *Castanea mollissima* | China | MK432615 | MK429885 | MK442939 | MK578075 | NA | NA |
| *C. myrtagena* | CBS 116843T | *Tibouchiina urvilleana* | USA | AY347363 | NA | NA | NA | NA | NA |
| *C. nitschkii* | CMW 10180T | *Eucalyptus globulus* | Ethiopia | AY347356 | NA | NA | NA | NA | NA |
| *C. nitschkii* | CMW 10184 | *Eucalyptus globulus* | Ethiopia | AY347355 | NA | NA | NA | NA | NA |
| *C. nivea* | MFLUCC 15-0860 | *Salix acutifolia* | Russia | KY417737 | KY417771 | KY417703 | KY417805 | NA | NA |
| *C. nivea* | CFCC 89641 | *Elaeagnus angustifolia* | Ningxia, China | KF765683 | KF765699 | KU711006 | KU710967 | KU710929 | KR045679 |
| *C. nivea* | CFCC 89643 | *Salix psammophila* | Shaanxi, China | KF765685 | KF765701 | NA | KU710968 | KP310863 | KP310829 |
| *C. notastroma* | NE_TFR5 | *Populus tremuloides* | USA | JX438632 | NA | NA | NA | JX438543 | NA |
| *C. notastroma* | NE_TFR8 | *Populus tremuloides* | USA | JX438633 | NA | NA | NA | JX438542 | NA |
| *C. ochracea* | CFCC 53164T | Cotoneaster sp. | Xinjiang, China | MK673060 | MK673090 | MK673030 | MK673001 | MK672949 | MK672976 |
| *C. oleicola* | CBS 144248T | *Olea europaea* | California, USA | MG971944 | NA | MG972098 | NA | MG971660 | MG971752 |
| *C. olivacea* | CFCC 53174 | *Prunus cerasifera* | Xinjiang, China | MK673058 | MK673088 | MK673028 | MK672999 | NA | MK672974 |
| *C. olivacea* | CFCC 53175 | *Prunus dulcis* | Xinjiang, China | MK673062 | MK673092 | MK673032 | MK673003 | NA | MK672978 |
| *C. olivacea* | CFCC 53176T | *Sorbus tianschanica* | Xinjiang, China | MK673068 | MK673098 | MK673038 | MK673008 | MK672955 | MK672984 |
| *C. olivacea* | CFCC 53177 | *Prunus virginiana* | Xinjiang, China | MK673071 | MK673101 | MK673041 | MK673011 | NA | MK672987 |
| *C. palm* | CXY 1276 | *Cotinus coggygria* | Beijing, China | JN402990 | NA | NA | NA | KJ781296 | NA |
| *C. palm* | CXY 1280T | *Cotinus coggygria* | Beijing, China | JN411939 | NA | NA | NA | KJ781297 | NA |
| *C. parakantschavelii* | MFLUCC 15-0857T | Populus × sibirica | Russia | KY417738 | KY417772 | KY417704 | KY417806 | NA | NA |
| *C. parakantschavelii* | MFLUCC 16-0575 | *Pyrus pyraster* | Russia | KY417739 | KY417773 | KY417705 | KY417807 | NA | NA |
| *C. parapersoonii* | T28.1T | *Prunus persica* | USA | AF191181 | NA | NA | NA | NA | NA |
| *C. parapistaciae* | CBS 144506T | *Pistacia vera* | California, USA | MG971804 | NA | MG971954 | NA | MG971519 | MG971669 |
| *C. parasitica* | MFLUCC 15-0507T | *Malus domestica* | Russia | KY417740 | KY417774 | KY417706 | KY417808 | NA | NA |
| *C. parasitica* | XJAU 2542-1 | Malus sp. | Xinjiang, China | MH798884 | MH798897 | NA | NA | MH813452 | NA |
| *C. parasitica* | CFCC 53171 | *Malus pumila* | Xinjiang, China | MK673061 | MK673091 | MK673031 | MK673002 | MK672950 | MK672977 |
| *C. parasitica* | CFCC 53172 | *Malus pumila* | Xinjiang, China | MK673069 | MK673099 | MK673039 | MK673009 | MK672956 | MK672985 |
| *C. parasitica* | CFCC 53173 | Berberis sp. | Xinjiang, China | MK673070 | MK673100 | MK673040 | MK673010 | MK672957 | MK672986 |
| *C. paratranslucens* | MFLUCC 15-0506T | Populus alba var. bolleana | Russia | KY417741 | KY417775 | KY417707 | KY417809 | NA | NA |
| *C. paratranslucens* | MFLUCC 16-0627 | *Populus alba* | Russia | KY417742 | KY417776 | KY417708 | KY417810 | NA | NA |
| *C. pavettae* | CBS 145562^T^ | *Pavetta revoluta* | South Africa | MK876386 | MK876427 | MK876457 | MK876483 | MK876497 | MK876503 |
| *C. piceae* | CFCC 52841T* | *Picea crassifolia* | Xinjiang, China | MH820398 | MH820391 | MH820406 | MH820395 | MH820402 | MH820387 |
| *C. piceae* | CFCC 52842* | *Picea crassifolia* | Xinjiang, China | MH820399 | MH820392 | MH820407 | MH820396 | MH820403 | MH820388 |
| *C. pingbianensis* | MFLUCC 18-1204T | Undefined wood | Yunnan, China | MK912135 | MK571763 | MN685817 | MN685826 | NA | NA |
| *C. pini* | CBS 197.42 | *Pinus sylvestris* | Switzerland | AY347332 | NA | NA | NA | NA | NA |
| *C. pini* | CBS 224.52T | *Pinus strobus* | USA | AY347316 | NA | NA | NA | NA | NA |
| *C. pistaciae* | CBS 144238T | *Pistacia vera* | California, USA | MG971802 | NA | MG971952 | NA | MG971517 | MG971667 |
| *C. platanicola* | MFLU 17-0327 | *Platanus hybrida* | Italy | MH253451 | MH253452 | MH253449 | MH253450 | NA | NA |
| *C. platycladi* | CFCC 50504T* | *Platycladus orientalis* | Yunnan, China | MH933645 | MH933679 | MH933552 | MH933610 | MH933516 | MH933581 |
| *C. platycladi* | CFCC 50505* | *Platycladus orientalis* | Yunnan, China | MH933646 | MH933680 | MH933553 | MH933611 | MH933517 | MH933582 |
| *C. platycladi* | CFCC 50506* | *Platycladus orientalis* | Yunnan, China | MH933647 | MH933681 | MH933554 | MH933612 | MH933518 | MH933583 |
| *C. platycladicola* | CFCC 50038T* | *Platycladus orientalis* | Gansu, China | KT222840 | MH933682 | MH933555 | MH933613 | MH933519 | MH933584 |
| *C. platycladicola* | CFCC 50039* | *Platycladus orientalis* | Gansu, China | KR045642 | KR045721 | KU711008 | KU710973 | KU710931 | KR045683 |
| *C. plurivora* | CBS 144239T | *Olea europaea* | California, USA | MG971861 | NA | MG972010 | NA | MG971572 | MG971726 |
| *C. phialidica* | MFLU 16-2442^T^ | *Alnus glutinosa* | Italy | MT177932 | MT177959 | NA | MT432209 | MT454014 | NA |
| *C. populicola* | CBS 144240T | *Populus deltoides* | California, USA | MG971891 | NA | MG972040 | NA | MG971601 | MG971757 |
| *C. populina* | CFCC 89644T | *Salix psammophila* | Shaanxi, China | KF765686 | KF765702 | KU711007 | KU710969 | KU710930 | KR045681 |
| *C. populinopsis* | CFCC 50032T | *Sorbus aucuparia* | Ningxia, China | MH933648 | MH933683 | MH933556 | MH933614 | MH933520 | MH933585 |
| *C. populinopsis* | CFCC 50033 | *Sorbus aucuparia* | Ningxia, China | MH933649 | MH933684 | MH933557 | MH933615 | MH933521 | MH933586 |
| *C. predappioensis* | MFLUCC 17-2458T | *Platanus hybrida* | Italy | MG873484 | MG873480 | NA | NA | NA | NA |
| *C. prunicola* | MFLU 17-0995T | Prunus sp. | Italy | MG742350 | MG742351 | MG742353 | MG742352 | NA | NA |
| *C. pruni-mume* | CFCC 53179 | *Prunus armeniaca* | Xinjiang, China | MK673057 | MK673087 | MK673027 | NA | MK672947 | MK672973 |
| *C. pruni-mume* | CFCC 53180T | *Prunus mume* | Xinjiang, China | MK673067 | MK673097 | MK673037 | MK673007 | MK672954 | MK672983 |
| *C. pruinopsis* | CFCC 50034T | *Ulmus pumila* | Shaanxi, China | KP281259 | KP310806 | KP310836 | KU710970 | KP310849 | KP310819 |
| *C. pruinopsis* | CFCC 50035 | *Ulmus pumila* | Jilin, China | KP281260 | KP310807 | KP310837 | KU710971 | KP310850 | KP310820 |
| *C. pruinopsis* | CFCC 53153 | *Ulmus pumila* | Beijing, China | MN854451 | MN854662 | MN850763 | MN850752 | MN850759 | MN861121 |
| *C. pruinosa* | CBS 201.42T | Syringa sp. | Switzerland | DQ243801 | NA | NA | NA | NA | NA |
| *C. pruinosa* | CFCC 50036 | *Syringa oblata* | Qinghai, China | KP310800 | KP310802 | KP310832 | NA | KP310845 | KP310815 |
| *C. pruinosa* | CFCC 50037 | *Syringa oblata* | Qinghai, China | MH933650 | MH933685 | MH933558 | NA | MH933522 | MH933589 |
| *C. pubescentis* | MFLUCC 18-1201T | *Quercus pubescens* | Forlì-Cesena, Italy | MK912130 | MK571758 | MN685812 | MN685821 | NA | NA |
| *C. punicae* | CBS 144244 | *Punica granatum* | California, USA | MG971943 | NA | MG972091 | NA | MG971654 | MG971798 |
| *C. quercicola* | MFLU 17–0881 | Quercus sp. | Italy | MF190128 | MF190074 | NA | NA | NA | NA |
| *C. quercicola* | MFLUCC 14-0867T | Quercus sp. | Italy | MF190129 | MF190073 | NA | NA | NA | NA |
| *C. rhizophorae* | MUCC302 | *Eucalyptus grandis* | Australia | EU301057 | NA | NA | NA | NA | NA |
| *C. ribis* | CFCC 50026 | *Ulmus pumila* | Qinghai, China | KP281267 | KP310813 | KP310843 | KU710972 | KP310856 | KP310826 |
| *C. ribis* | CFCC 50027 | *Ulmus pumila* | Qinghai, China | KP281268 | KP310814 | KP310844 | NA | KP310857 | KP310827 |
| *C. ribis* | CBS 187.36 | *Ribes rubrum* | Netherlands | DQ243810 | NA | NA | NA | NA | NA |
| *C. rosae* | MFLU 17-0885 | *Rosa canina* | Italy | MF190131 | MF190076 | NA | NA | NA | NA |
| *C. rosicola* | CF 20197024T | Rosa sp. | Tibet, China | MK673079 | MK673109 | MK673049 | MK673019 | MK672965 | MK672995 |
| *C. rosigena* | MFLUCC 18-0921^T^ | Rosa sp. | Russia | MN879872 | MN879873 | NA | NA | NA | NA |
| *C. rostrata* | CFCC 89909T | *Salix cupularis* | Gansu, China | KR045643 | KR045722 | KU711009 | KU710974 | KU710932 | KR045684 |
| *C. rostrata* | CFCC 89910 | *Salix cupularis* | Gansu, China | KR045644 | KR045723 | KU711010 | KU710975 | KU710933 | NA |
| *C. rusanovii* | MFLUCC 15-0853 | Populus × sibirica | Russia | KY417743 | KY417777 | KY417709 | KY417811 | NA | NA |
| *C. rusanovii* | MFLUCC 15-0854T | *Salix babylonica* | Russia | KY417744 | KY417778 | KY417710 | KY417812 | NA | NA |
| *C. salicacearum* | MFLUCC 16-0576 | Populus nigra var. italica | Russia | KY417741 | KY417775 | KY417707 | KY417809 | NA | NA |
| *C. salicacearum* | MFLUCC 16-0587 | *Prunus cerasus* | Russia | KY417742 | KY417776 | KY417708 | KY417810 | NA | NA |
| *C. salicacearum* | MFLUCC 15-0861 | Salix × fragilis | Russia | KY417745 | KY417779 | KY417711 | KY417813 | NA | NA |
| *C. salicacearum* | MFLUCC 15-0509T | *Salix alba* | Russia | KY417746 | KY417780 | KY417712 | KY417814 | NA | NA |
| *C. salicicola* | MFLUCC 15-0866 | *Salix alba* | Russia | KY417749 | KY417783 | KY417715 | KY417817 | NA | NA |
| *C. salicicola* | MFLUCC 14-1052T | *Salix alba* | Russia | KU982636 | KU982635 | KU982637 | NA | NA | NA |
| *C. salicina* | MFLUCC 15-0862T | *Salix alba* | Russia | KY417750 | KY417784 | KY417716 | KY417818 | NA | NA |
| *C. salicina* | MFLUCC 16-0637 | Salix × fragilis | Russia | KY417751 | KY417785 | KY417717 | KY417819 | NA | NA |
| *C. schulzeri* | CFCC 50040 | *Malus domestica* | Ningxia, China | KR045649 | KR045728 | KU711013 | KU710980 | KU710936 | KR045690 |
| *C. schulzeri* | CFCC 50042 | *Malus pumila* | Gansu, China | KR045650 | KR045729 | KU711014 | KU710981 | KU710937 | KR045691 |
| *C. sibiraeae* | CFCC 50045T | *Sibiraea angustata* | Gansu, China | KR045651 | KR045730 | KU711015 | KU710982 | KU710938 | KR045692 |
| *C. sibiraeae* | CFCC 50046 | *Sibiraea angustata* | Gansu, China | KR045652 | KR045731 | KU711015 | KU710983 | KU710939 | KR045693 |
| *C. sophorae* | CFCC 50047 | *Styphnolobium japonicum* | Shanxi, China | KR045653 | KR045732 | KU711017 | KU710984 | KU710940 | KR045694 |
| *C. sophorae* | CFCC 50048 | *Magnolia grandiflora* | Shanxi, China | MH820401 | MH820394 | MH820409 | MH820397 | MH820405 | MH820390 |
| *C. sophorae* | CFCC 89598 | *Styphnolobium japonicum* | Gansu, China | KR045654 | KR045733 | KU711018 | KU710985 | KU710941 | KR045695 |
| *C. sophoricola* | CFCC 89596 | *Styphnolobium japonicum* var. *pendula* | Gansu, China | KR045656 | KR045735 | KU711020 | KU710987 | KU710943 | KR045697 |
| *C. sophoricola* | CFCC 89595T | *Styphnolobium japonicum* var. *pendula* | Gansu, China | KR045655 | KR045734 | KU711019 | KU710986 | KU710942 | KR045696 |
| *C. sophoriopsis* | CFCC 89600T | *Styphnolobium japonicum* | Gansu, China | KR045623 | KP310804 | KU710992 | KU710951 | KU710915 | KP310817 |
| *C. sorbi* | MFLUCC 16-0631T | *Sorbus aucuparia* | Russia | KY417752 | KY417786 | KY417718 | KY417820 | NA | NA |
| *C. sorbicola* | MFLUCC 16-0584T | *Acer pseudoplatanus* | Russia | KY417755 | KY417789 | KY417721 | KY417823 | NA | NA |
| *C. sorbicola* | MFLUCC 16-0633 | *Cotoneaster melanocarpus* | Russia | KY417758 | KY417792 | KY417724 | KY417826 | NA | NA |
| *C. sorbina* | CF 20197660T | *Sorbus tianschanica* | Xinjiang, China | MK673052 | MK673082 | MK673022 | NA | MK672943 | MK672968 |
| *C. spiraeae* | CFCC 50049T | *Spiraea salicifolia* | Gansu, China | MG707859 | MG707643 | MG708196 | MG708199 | NA | NA |
| *C. spiraeae* | CFCC 50050 | *Spiraea salicifolia* | Gansu, China | MG707860 | MG707644 | MG708197 | MG708200 | NA | NA |
| *C. spiraeicola* | CFCC 53138T | *Spiraea salicifolia* | Beijing, China | MN854448 | MN854659 | NA | MN850749 | MN850756 | MN861118 |
| *C. spiraeicola* | CFCC 53139 | *Tilia nobilis* | Beijing, China | MN854449 | MN854660 | NA | MN850750 | MN850757 | MN861119 |
| *C. tamaricicola* | CFCC 50507 | *Rosa multifolora* | Yunnan, China | MH933651 | MH933686 | MH933559 | MH933616 | MH933525 | MH933587 |
| *C. tamaricicola* | CFCC 50508T | *Tamarix chinensis* | Yunnan, China | MH933652 | MH933687 | MH933560 | MH933617 | MH933523 | MH933588 |
| *C. tanaitica* | MFLUCC 14-1057T | *Betula pubescens* | Russia | KT459411 | KT459412 | KT459413 | NA | NA | NA |
| *C. thailandica* | MFLUCC 17-0262T | *Xylocarpus moluccensis* | Thailand | MG975776 | MH253463 | MH253459 | MH253455 | NA | NA |
| *C. thailandica* | MFLUCC 17-0263T | *Xylocarpus moluccensis* | Thailand | MG975777 | MH253464 | MH253460 | MH253456 | NA | NA |
| *C. tibetensis* | CF 20197026 | Cotoneaster sp. | Tibet, China | MK673076 | MK673106 | MK673046 | MK673016 | MK672962 | MK672992 |
| *C. tibetensis* | CF 20197029 | Cotoneaster sp. | Tibet, China | MK673077 | MK673107 | MK673047 | MK673017 | MK672963 | MK672993 |
| *C. tibetensis* | CF 20197032T | Cotoneaster sp. | Tibet, China | MK673078 | MK673108 | MK673048 | MK673018 | MK672964 | MK672994 |
| *C. tibouchinae* | CPC 26333T | *Tibouchina semidecandra* | France | KX228284 | KX228335 | NA | NA | NA | NA |
| *C. translucens* | CXY 1351 | *Populus davidiana* | Inner Mongolia, China | KM034874 | NA | NA | NA | NA | KM034895 |
| *C. ulmi* | MFLUCC 15-0863T | *Ulmus minor* | Russia | KY417759 | NA | NA | NA | NA | NA |
| 1. *ulmicola* | MFLUCC 18-1227T | *Ulmus pumila* | Russia | MH940220 | MH940218 | MH940216 | NA | NA | NA |
| *C. valsoidea* | CMW 4309T | *Eucalyptus grandis* | Indonesia | AF192312 | NA | NA | NA | NA | NA |
| *C. valsoidea* | CMW 4310 | *Eucalyptus grandis* | Indonesia | AF192312 | NA | NA | NA | NA | NA |
| *C. variostromatica* | CMW 6766T | *Eucalyptus globulus* | Australia | AY347366 | NA | NA | NA | NA | NA |
| *C. variostromatica* | CMW 1240 | *Eucalyptus grandis* | South Africa | AF260263 | NA | NA | NA | NA | NA |
| *C. variostromatica* | PPRI5297 | *Eucalyptus grandis* | South Africa | AF260264 | NA | NA | NA | NA | NA |
| ***C. verrucosa*** | **CFCC 53157T*** | *Platycladus orientalis* | Beijing, China | MW418408 | MW418420 | NA | MW422911 | MW422923 | MW422935 |
| ***C. verrucosa*** | **CFCC 53158*** | *Platycladus orientalis* | Beijing, China | MW418410 | MW418422 | MW422901 | MW422913 | MW422925 | MW422937 |
| ***C. verrucosa*** | **CFCC 54369*** | *Platycladus orientalis* | Beijing, China | MW418409 | MW418421 | NA | MW422912 | MW422924 | MW422936 |
| ***C. verrucosa*** | **CFCC 54370*** | *Platycladus orientalis* | Beijing, China | MW418411 | MW418423 | MW422902 | MW422914 | MW422926 | MW422938 |
| *C. vinacea* | CBS 141585T | *Vitis interspecific* hybrid ‘Vidal’ | USA | KX256256 | NA | NA | NA | KX256277 | KX256235 |
| *C. viridistroma* | CBS 202.36^T^ | *Cercis canadensis* Castigl. | USA | MN172408 | MN172388 | NA | NA | MN271853 | NA |
| *C. viticola* | Cyt2 | *Vitis interspecific* hybrid ‘Frontenac’ | USA | KX256238 | NA | NA | NA | KX256259 | KX256217 |
| *C. viticola* | CBS 141586T | *Vitis vinifera* ‘Cabernet Franc’ | USA | KX256239 | NA | NA | NA | KX256260 | KX256218 |
| *C. xinjiangensis* | CFCC 53182 | Rosa sp. | Xinjiang, China | MK673064 | MK673094 | MK673034 | MK673004 | MK672951 | MK672980 |
| *C. xinjiangensis* | CFCC 53183T | Rosa sp. | Xinjiang, China | MK673065 | MK673095 | MK673035 | MK673005 | MK672952 | MK672981 |
| *C xinglongensis* | CFCC 52458 | *Castanea mollissima* | China | MK432622 | MK429892 | MK442946 | MK578082 | NA | NA |
| *C xinglongensis* | CFCC 52459 | *Castanea mollissima* | China | MK432623 | MK429893 | MK442947 | MK578083 | NA | NA |
| *C. xylocarpi* | MFLUCC 17-0251T | *Xylocarpus granatum* | Thailand | MG975775 | MH253462 | MH253458 | MH253454 | NA | NA |
| *Diaporthe vaccinii* | CBS 160.32 | *Vaccinium macrocarpon* | USA | KC343228 | NA | JQ807297 | NA | KC343954 | KC344196 |

^1^ Acronyms: ATCC: American Type Culture Collecton, Virginia, USA; BBH: BIOTEC Bangkok Herbarium, National Science and Technology Development Agency, Thailand; CBS: Westerdijk Fungal Biodiversity Institute (CBS-KNAW Fungal Biodiversity Centre), Utrecht, The Netherlands; CFCC: China Forestry Culture Collection Centre, Beijing, China; CMW: Culture collection of Michael Wingfield, University of Pretoria, South Africa; CPC: Culture collection of Pedro Crous, The Netherlands; IMI: Culture collection of the International Mycological Institute, CABI Bioscience, Egham, Surrey, UK; MFLU: Mae Fah Luang University herbarium, Thailand; MFLUCC: Mae Fah Luang University Culture Collection, Thailand; MUCC: Murdoch University Culture Collection, Perth, Australia; NE: Gerard Adams collections, University of Nebraska, Lincoln NE, USA; PPRI: Culture collection of the Plant Protection Research Institute, Agriculture Research Center, Pretoria, South Africa; XJAU: Xinjiang Agricultural University, Xinjiang, China; NA: not applicable. All the new isolates used in this study are in bold and the type materials are marked with T. All the *Cytospora* species listed from coniferous trees in China are marked with *.
